# CBD hydroxyquinone photo-isomerises to a highly reactive intermediate

**DOI:** 10.1038/s41598-023-33815-7

**Published:** 2023-04-28

**Authors:** Brodie. J. Thomson, Summer Hanna, Adrian Schwarzenberg, Pirouz Kiani, Dan Bizzotto, Pierre Kennepohl, Ashley Davies, Markus Roggen, Glenn M. Sammis

**Affiliations:** 1 Department of Chemistry, University of British Columbia, Vancouver, Canada; 2 Group Research and Development Centre, BAT Investments Limited, Southampton, UK; 3 Department of Chemistry, University of Calgary, Calgary, Canada; 4 DELIC Labs, Vancouver, Canada

**Keywords:** Physical chemistry, Computational chemistry, Analytical chemistry, NMR spectroscopy, Density functional theory

## Abstract

The legalisation of hemp has led to wide commercial availability of cannabidiol (CBD)-containing products. Here we show that the CBD-hydroxyquinone (HU-331), a readily formed oxidation product and common impurity in CBD isolates, undergoes a previously unknown photo-isomerisation to produce a highly reactive intermediate in solution. Studies supported by calculations indicate that this intermediate rapidly reacts with oxygen to form a multitude of cannabinoid products. The purple colour observed in light-aged CBD-containing solutions is largely due to the anions of these by-products and is not significantly due to the HU-331 anion. Our findings suggest that these uncharacterized cannabinoid derivatives can be present in CBD-containing e-liquids and solutions that have been stored under ambient light conditions, calling for quality control processes that manage HU-331 contamination.

## Introduction

**Figure 1:**
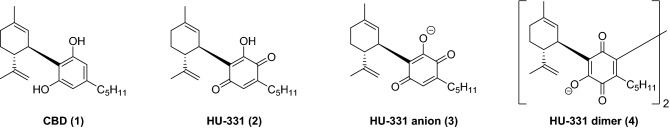
Structures of cannabidiol (CBD) and its formed degradants in the Beam test. CBD (**1**), HU-331 (**2**), HU-331 anion (**3**) and HU-331 anion dimer (**4**).

The legalization of *Cannabis sativa* plants containing very low levels of the intoxicating chemical delta-9-tetrahydrocannabinol (THC) in countries such as the United Kingdom and United States has sparked renewed interest in the health benefits of non-intoxicating cannabinoids^1–3^. In particular, cannabidiol (CBD, **1**, Fig. 1) has become increasingly popular, with numerous data suggesting its potential with respect to anti-inflammatory, neuroprotective, anxiolytic, analgesic, anti-arthritic and antitumor properties^4^. As a result, CBD (**1**) is widely available in various product formats including tinctures, oils, lozenges and vape e-liquids, and global sales of CBD (**1**) are predicted to exceed US $19 billion by 2025^5^. However, the fragmented legal status and rapid market entry has preceded the regulation of some of these consumer goods. A recurring concern within the scientific literature is the lack of quality control enforced on such production, demonstrated through differences between measured CBD (**1**) concentrations and levels stated on the packaging of some of the currently available products^6–8^.

Another area that has not been considered in detail by regulators is the degradation of CBD-containing products. Anecdotal evidence suggests that some CBD (**1**) solutions change colour on storage^9–12^. For example, CBD-containing e-liquids reportedly turn from colourless to a deep purple colour^12^, suggesting degradation. A thorough understanding of the factors that lead to decomposition and the identity of the resulting products is vital to enable rigorous safety assessments. Although CBD (**1**) in solution is clearly susceptible to decomposition^7,13–17^, to our knowledge, no studies have attempted to identify the degradation products responsible for the discolouration. As a result, the potential health risks that such impurities may pose are unknown. A cannabinoid degradation process known to produce a deep purple colour is the Beam test, a classical colourimetric test established for the identification of hashish^18^. The test involves treating a sample with 5% ethanolic potassium hydroxide (KOH); development of a purple solution confirms the presence of cannabinoids, specifically CBD (**1**) and the minor hashish components cannabigerol (CBG) and cannabidiolic acid (CBDA)^19^. Seminal studies by Mechoulam’s group determined that the colour change was due to the formation of HU-331 anion (**3**) and anionic dimer (**4**) under the basic conditions of the test^20,21^. More recently, purple colouration of some CBD solutions has been linked to HU-331 (**2**) formation^22^.

We set out to investigate anecdotal reports of the visible degradation of CBD-containing solutions given the increasing availability of CBD (**1**) and dearth of information on its breakdown products. Unexpectedly, we found that the CBD oxidation product HU-331 (**2**) is photochemically unstable, forming a previously unknown and extremely reactive hydroxyquinol intermediate. In the presence of oxygen, this hydroxyquinol rapidly reacts to form a multitude of purple-coloured cannabinoid by-products. We discuss our findings in terms of the mechanistic pathway and implications for the quality control of CBD-containing products, specifically those in solution format.

## Results

### CBD solution turns purple due to a photochemical process

Given the recent growth of CBD-containing e-cigarette products^23^, we began investigations into the stability of CBD (**1**) solutions by carrying out experiments on samples of CBD (**1**) e-liquid stored in different conditions with a particular focus on tracking HU-331 (**2**) concentration. The CBD (**1**) e-liquid was prepared from 65% propylene glycol (PG), 30% vegetable glycerine (VG) and 5% CBD (**1**) by mass. Liquid chromatography mass spectrometry analysis (HPLC–MS/MS) showed that the CBD (**1**) oxidation product HU-331 (**2**) was present in the fresh e-liquid analysed immediately after CBD dissolution (0.05 ± 0.04 µg/g). HU-331 (**2**) is reported as an orange solid^24^, but, at the low concentration detected, the e-liquid appeared colourless (Supplementary Fig. S1). We confirmed that HU-331 (**2**) was introduced via the raw CBD (**1**) isolate, which contained trace amounts of HU-331 (**2**, 0.86 ± 0.11 µg/g). It is important to note that HU-331 (**2**) is not routinely tested for in CBD-containing products, and its presence in raw material suggests that CBD (**1**) oxidation occurs during production.

Aliquots of the fresh e-liquid were stored in sealed glass vials undisturbed under ambient laboratory conditions (~ 25 °C, ~ 60% relative humidity) either in the dark or exposed to natural light/dark cycles. After 10 weeks, the samples stored in the dark had visibly turned from colourless to yellow-orange, consistent with air oxidation of CBD (**1**) to HU-331 (**2**)^25^ whereas those stored under natural light/dark cycles had turned violet/purple (Supplementary Fig. S1) in line with anecdotal reports^16,18^. The concentration of HU-331 (**2**) had increased in both storage conditions and was approximately fourfold higher in the orange dark-stored samples (116.03 µg/g) than in the purple light-stored samples (25.04 µg/g). Thus, storage of CBD (**1**) e-liquid for 10 weeks led to visible, but potentially different, changes under dark and natural light conditions. The mechanism of the oxygen-promoted CBD (**1**) oxidation to HU-331 (**2**) in e-liquids likely differs to the oxidation under the Beam test conditions only in reaction rate. The key step is the single electron oxidation of the resorcinol moiety^26^. Under basic (Beam test) conditions, the ring is already deprotonated and, thus, the oxidation proceeds more readily. Under neutral (e-liquid) conditions, the resorcinol is mostly protonated and results a slow conversion to HU-331 (**2**).

Given the colourimetric similarity between our light-stored samples and the reported HU-331 anion (**3**)^21,22^, we considered whether the process responsible for the purple colour in the highly basic Beam test may be taking place in the light-exposed neutral CBD e-liquid. To test this possibility, we first synthesized and isolated components of the classic test as reagents and reference compounds. We prepared HU-331 (**2**), an orange solid, by a published protocol^27^. We then isolated HU-331 anion **3** directly from the deep purple solution formed after the addition of KOH to CBD (**1**) (i.e., Beam test)^20,21^. The major purple component was confirmed as the potassium salt of anion **3** by nuclear magnetic resonance (NMR) spectroscopy, including ^1^H-NMR, ^13^C-NMR, HSQC and HMBC spectroscopic analysis (Supplementary Figs. S4–S7).

**Figure 2:**
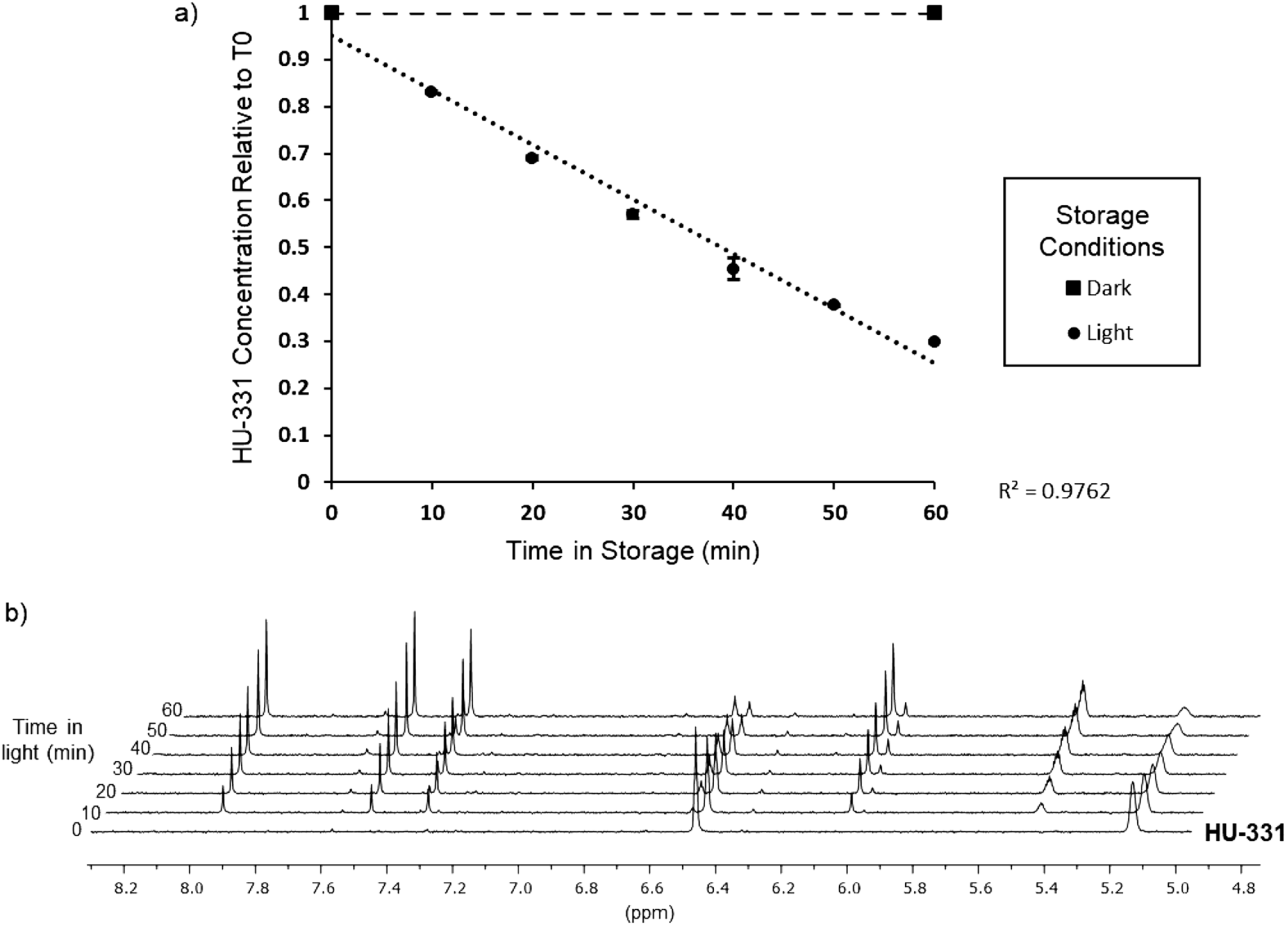
Effect of visible light on HU-331 concentrations. (**a**) Changes in concentration of HU-331 (6 mM in DMSO-*d*_*6*_) stored in the dark (squares) or exposed to 6500 K fluorescence (circles) for up to 60 min. HU-331 concentration was measured by ^1^H-NMR. (**b**) ^1^H NMR of HU-331 samples degraded by white light over time.

Using the synthesised HU-331 (**2**), we looked more closely at the effects of light exposure on solutions of HU-331 (**2**). We used dimethyl sulfoxide (DMSO) as the solvent due to its ability to dissolve both neutral and ionic species. Samples of HU-331 (**2**) in DMSO-*d*_*6*_ (6 mM) spiked with a 1,4-dinitrobenzene internal standard were either foil-wrapped and stored in the dark at room temperature or exposed to a 6500 K fluorescent bulb for durations of 10–60 min before immediate analysis by ^1^H-NMR. The sample stored in the dark for 60 min showed no change in HU-331 (**2**) concentration, whereas the light-exposed samples showed a marked decrease that directly correlated with exposure time (Fig. 2a). After 60 min, the concentration of HU-331 (**2**) had dropped by approximately 70%, while numerous unknown peaks appeared in the NMR spectrum (Fig. 2b). Importantly, none of the unknown peaks could be attributed to HU-331 anion **3** (Supplementary Figs. S8–S15). Collectively, these preliminary observations indicate that HU-331 (**2**) is photochemically unstable, rapidly breaking down on exposure to visible light to form a purple solution containing multiple unknown cannabinoids, but not HU-331 anion **3**.

### HU-331 photochemically isomerises to a highly reactive hydroxyquinol

Based on the above experiments, decomposition of HU-331 (**2**) is likely to be involved in the colour changes occurring in CBD (**1**) solution stored in natural light. This hypothesis is consistent with the UV–visible absorbance spectra of both HU-331 (**2**), which absorbs light with a wavelength maximum of 413 nm, and CBD (**1**), which does not absorb in the visible range (Supplementary Fig. S16).

We used this difference in visible-light absorption to probe the degradation process further. We selectively photoexcited 3 mM solutions of CBD (**1**) or HU-331 (**2**) in deuterated DMSO at 448 nm, a wavelength that avoided overlap with the absorbance spectrum of CBD (**1**), and monitored the reaction by NMR spectroscopy. As expected, no spectral changes were observed after irradiation of CBD (**1**) with 448-nm light (Supplementary Fig. S18). By contrast, 15-min light irradiation of HU-331 in deuterated DMSO led to its complete conversion to a previously unidentified cannabinoid species (Supplementary Fig. S19). HU-331 (**2**) formed the unidentified cannabinoid in the presence of CBD (**1**) and regardless of relative CBD (**1**) concentration, suggesting that the cannabinoid is formed through an intramolecular pathway (Supplementary Figs. S20 and S21).

The unknown species was observed in various solvent systems, including isopropyl alcohol and the common e-liquid solvent PG (Supplementary Figs. S22 and S23). It was colourless in solution, unstable in air and rapidly degraded during attempts to isolate it. Exposure to oxygen led to the appearance of a red colouration, spreading from the liquid–air interface within 24 h, followed by complete degradation of the species within 48 h, coincidental with the solution turning purple (Supplementary Fig. S33); this degradation was observed in both the presence and absence of CBD (**1**) (Supplementary Figs. S24 and S25).

**Figure 3:**
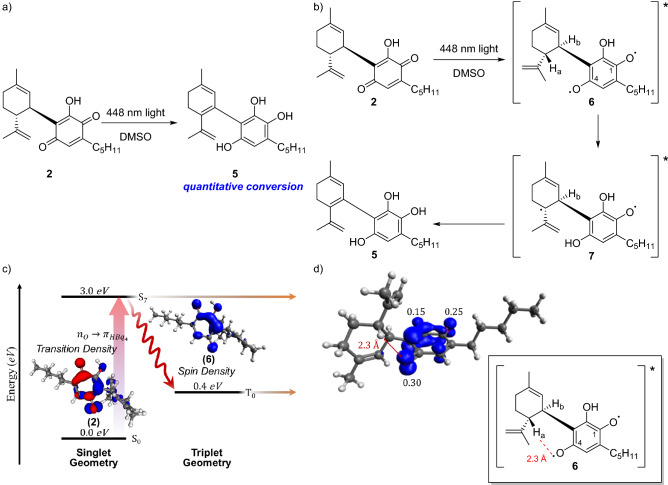
Mechanism of the photo-induced HU-331 light isomerisation. Formation of highly reactive cannabinoid hydroxyquinol (**5**). (**a**) Photochemical synthesis of hydroxyquinol (**5**) from HU-331 (**3**). (**b**) Proposed mechanism of formation. (**c**) Energies of the ground state and key excited states of HU-331. (**d**) Molecular model showing the key distance between the hydroxyquinol radical and the C–H on the limonene moiety.

However, the novel species was stable under oxygen-free conditions (Supplementary Fig. S26), allowing for a full structural determination. Extensive spectroscopic (^1^H-NMR, ^13^C-NMR, HSQC and HMBC) and spectrometric analysis indicated that the cannabinoid derivative was the hydroxyquinol **5** (C_21_H_27_O_3_; Fig. 3a and Supplementary Figs. S28–S31), an isomerisation product of HU-331 (**2**) that, to our knowledge, has yet to be reported in any study of either CBD (**1**) degradation or hydroquinone chemistry. The closest similarity to this intermediate is the enol tautomer that forms on the photochemical excitation of 5-methyl-1,4-napthoquinone^28^.

We probed the initial steps of the photochemical formation of **5** by using density functional theory (DFT) methods, which suggested that photoexcitation of the hydroxyquinone core leads to the reactive diradical intermediate **6** (Fig. 3b). Time-dependent (TD)-DFT calculations show that visible-light absorption results in a high-energy singlet diradical excited state, which has large spin density contributions on all three oxygen atoms of the hydroxyquinone (Fig. 3c). The corresponding relaxed triplet diradical is more stable, but still displays localized spin density on each of the hydroxyquinone oxygen atoms. The preferential localization of spin density on the terminal oxygen atoms suggests that the radical intermediate is susceptible to further radical chemistry.

Because our above experiments showed that the photochemical process is concentration-independent and does not lead to a photo-degradation of CBD (**1**), further reactivity of the diradical species is probably dominated by an intramolecular process (**6**–**7**, Fig. 3b). The formation of **7** from **6** must therefore proceed by either direct hydrogen atom transfer (HAT) or proton-coupled electron transfer (PCAT). Given the structural constraints, it seems most likely that the intramolecular reactivity of **6** is facilitated by the close proximity of the oxygen atom with the largest spin density (4-O) to H_a_ on the limonene group (r_OH_ ≈ 2.3 Å, Fig. 3d). HAT and/or PCAT via this pathway would proceed via a favourable six-membered transition state. Alternative conformations of the diradical species do not permit other protons in the molecule, such as H_b_, to be accessed as easily from either 4-O or 1-O (**6**, Fig. 3b). Intermolecular hydrogen abstraction processes have been observed for other benzoquinones such as chloranil^29^, and 2,3-dichloro-5,6-dicyanoquinone (DDQ)^30^. After formation of **7**, several pathways to hydroxyquinol **3** (Fig. 3b), including both HAT and oxidation/elimination mechanisms, are feasible.

Collectively, these observations indicate that selective photoexcitation of the hydroxyquinone tethered to the easily oxidized limonene moiety leads to quantitative conversion of HU-331 (**2**) to the hydroxyquinol **5** via intramolecular radical processes. Given that **5** is formed as the sole product in solution upon photoexcitation of HU-331, we propose that the formation of **5** is the critical species through which the large array of decomposition products observed in MS analysis of light-stored CBD (**1**) solutions is generated (Supplementary Fig. S32). Because this photoexcitation readily occurs in visible light, the reaction may be proceeding in ambiently stored CBD (**1**) solution products that contain HU-331 (**2**) as a common impurity.

### Cascading reactions of the hydroxyquinol

**Figure 4:**
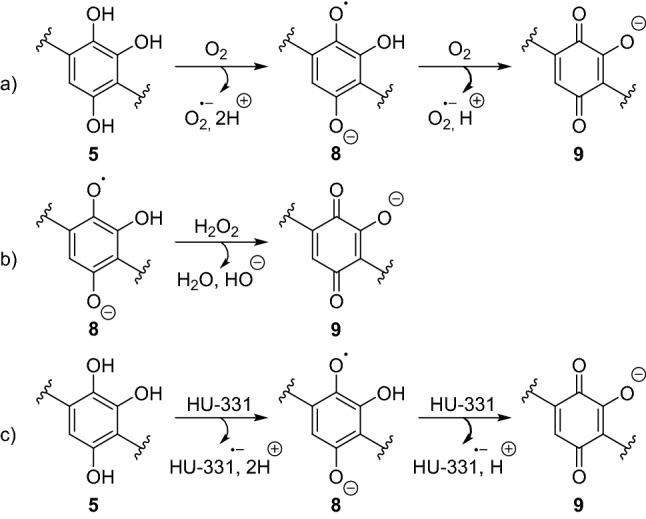
Formation and reaction of hydroxyquinol **5** and related products in CBD solutions.

To confirm that hydroxyquinol **5** is the key intermediate that results in the formation of numerous cannabinoid derivatives in light-stored CBD (**1**) solutions, we examined its reactivity. ^1^H-NMR spectroscopic analysis suggested that a complex mixture of compounds, in addition to hydrogen peroxide, formed after air exposure and decomposition of the hydroxyquinol (Supplementary Figs. S34 and S35). The formation of hydrogen peroxide is consistent with previously reported autoxidation of hydroxyquinols in the presence oxygen (Fig. 4a)^31^. Superoxide is released as a by-product and probably disproportionates to hydrogen peroxide in the protic medium^32^. In our experiments, the presence of water or the low pK_a_ (≈ 4.3) of hydroxyquinones^33^, is likely to supplement this reaction, resulting in a high concentration of deprotonated quinone **9**, which accounts for the observed purple colour.

A hydrogen peroxide reduction by semiquinone **8** is also possible and would similarly afford quinone **9** (Fig. 4b) when oxygen concentrations are low^29^. The hydroxide by-product would not only perpetuate the cycle of CBD (**1**) degradation to HU-331 (**2**), but also lead to an array of compounds from the reaction with **5**^34^. Solutions that contain higher concentrations of HU-331 (**2**) relative to oxygen or hydrogen peroxide would also be prone to cross oxidation with HU-331 (**2**), affording similar quinone anion products (Fig. 4c)^32^.

The release of reactive oxygen species such as superoxide is likely to affect CBD (**1**) concentration over time, owing to the antioxidant behaviour of CBD (**1**)^35,36^. Thus, an increasingly complex mixture of products will result from the reactions highlighted in Fig. 4. This hypothesis is supported by the significant number of products proposed to include both the core hydroxyquinone and limonene moieties observed by LC/MS analysis of solutions of CBD (**1**)/HU-331 (**2**) aged in light (Supplementary Figs. S36, Table S2)**.**

**Figure 5:**
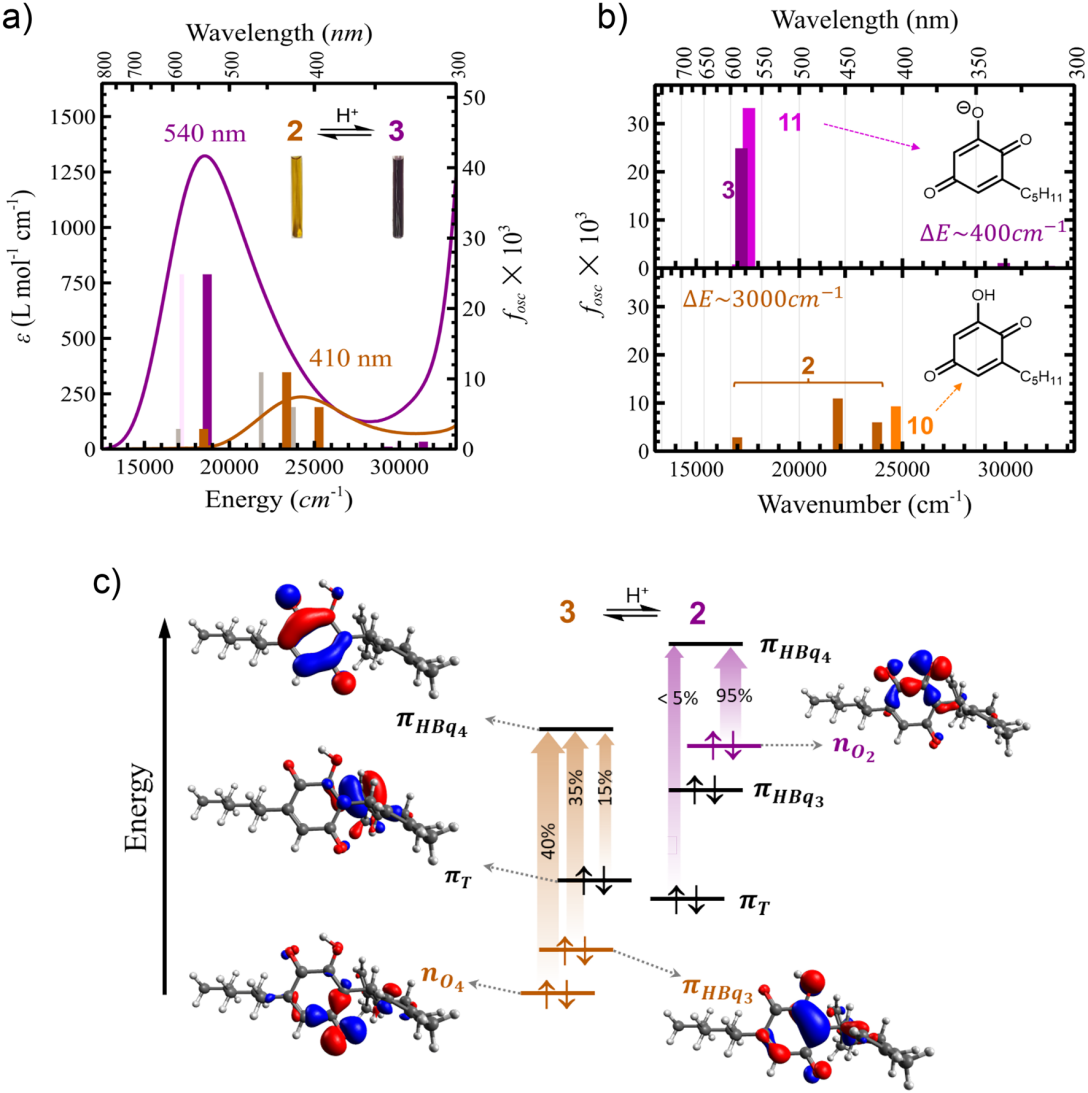
Purple colour origin in 2-hydroxyquinone anions. (**a**) Electronic absorption spectra in molar absorptivity ($\varepsilon$) and TD-DFT-computed oscillator strengths (f_osc_) for **2** (orange) and its anion **3** (purple) in isopropanol. The simulated data were well matched to the experimental spectra by adding a small shift of + 1500 cm^–1^. (**b**) Effect of the limonene group on the electronic absorption spectra based on TD-DFT calculations for both the neutral species (**2** vs **10**) and their anions (**3** vs **11**); the limonene group has a significant impact on the spectra of the neutral species but not the anions. (**c**) Simplified representation of the dominant contributions to the most intense visible absorption bands in **2** and **3**. Contours maps of the contributing canonical molecular orbitals (0.05 au isodensity) are shown for the neutral species.

An unexpected observation was the highly consistent purple colour between the oxidation products of the hydroxyquinol **5** and HU-331 anion **3**, because substituent effects would be expected to influence the UV–visible absorption profile. We therefore explored the electronic absorption spectra of these species, together with the simpler 2-hydroxyquinone of olivetol (**10**, R_1_ = H, R_2_ = C_5_H_11_), by TD-DFT (Fig. 5). We first checked that the visible-light absorption spectra calculated for both HU-331 (**2**) and HU-331 anion (**3**) correspond closely to the experimental data (Fig. 5a), confirming that a minimal systematic shift of + 1500 cm^–1^ leads to near-perfect agreement with the experimental spectra.

HU-331 (**2**) shows weak absorption at 410 nm ($\varepsilon$_410_ ≈ 250 L mol^–1^ cm^–1^) in the visible region, giving rise to its dull orange colour. By contrast, the hydroxyquinone of olivetol (**10**, Fig. 5b) is a nearly colourless hydroxyquinone that exhibits both weaker and higher energy absorption in the visible region relative to HU-331 (Fig. 5b, bottom). The difference in the absorption spectra between **2** and **10** is directly linked to the influence of the limonene moiety. In the absence of the limonene moiety, visible light absorption (< 400 nm) results from excitation of an oxygen-centered lone pair electron in the hydroxybenzoquinone moiety to the first unoccupied $\pi$ orbital (*n*_O_ → $\pi$_HBq4_).

This differs from **2**, where this absorption splits into multiple contributions due to direct coupling between the donor orbital and filled orbitals on both the hydroxyquinone $\pi$ system ($\pi$_HBq3_) and the terminal alkene of the limonene moiety ($\pi$_*T*_) (Fig. 5c, left). This mixing is substantial due to both appropriate energy matching and reasonable overlap among the different contributors. The limonene moiety has a significant impact on the photochemical properties of the hydroxybenzoquinone in its neutral form both by (i) lowering the energy of the photochemically active excited state and (ii) providing a properly positioned hydrogen atom that results in efficient photochemical reactivity. We postulate that photo-isomerisation of hydroxyquinones has not previously been observed for this reason.

The situation differs substantially in anion **3**, both in the intensity of the transition and its significantly lower energy (versus the neutral species). Deprotonation of the 2-hydroxy group changes the energetics such that the lone pairs on this oxygen are now at much higher energy and the *n*_O_ → $\pi$_HBq4_ transition now comes from the oxygen at the 2-position rather than that at the 4-position. This large energy shift in the donor orbital decouples this intense charge transfer transition with both the hydroxyquinone system and the limonene substituent (Fig. 5c, right). This decoupling explains the observed insensitivity of the absorption spectrum of deprotonated hydroxyquinones to the nature of substituents around the ring. Is should be noted that, when deprotonated under beam test conditions, olivetol shows the characteristic purple colour and has a UV–visible spectrum nearly identical to that of HU-331 anion (Supplementary Fig. S37). Correspondingly, we observe essentially no difference in the computed spectra of **11** and **3** (Fig. 5b, top). Thus, we propose that, in solution and under light exposure, other cannabinoids that possess a resorcinol ring will oxidise and form purple anions similar to that of HU-331 due to the presence of their respective hydroxyquinones.

## Discussion

Using a combination of experimental studies, we have shown that the CBD (**1**) oxidation product HU-331 (**2**) is photochemically unstable and forms a previously unknown and extremely reactive hydroxyquinol intermediate in solution. In the presence of oxygen and light—conditions under which many CBD-containing consumer products may be stored—the hydroxyquinol rapidly reacts to form a multitude of cannabinoid products, including purple-coloured quinone anions. It should be noted here that our experimental studies were performed on CBD-containing solutions, hence the results cannot necessarily be translated to CBD (**1**) products in solid format.

Given the variability in regulations that govern recreational CBD-products, combined with the predicted increase in the global CBD market^5^, these findings provide important new information that can help inform product standards. Industry-standardised laboratory testing that enables identification and quantification of the multitude of CBD (**1**) degradation products should be mandatory for all CBD-products to help protect consumer health and safety. In particular, manufacturing processes that limit the formation and subsequent degradation of HU-331 (**2**) to manage contaminant levels should be implemented, given that the potential health risks of the resulting impurities remain unknown. In this regard, it has been reported that HU-331 (**2**) itself has promising anticancer properties and its photo-instability—as well as that of other cannabinoid hydroxyquinones—should be considered when assessing the potential use of these compounds^25,37^.

We consider that the light-induced degradation pathway of HU-331 (**2**) will apply to other resorcinol-based cannabinoids, with the potential formation of many new compounds. Studying these pathways and compounds will be fundamental in ensuring that cannabinoid-containing products are designed to prevent such chemistry from occurring and will be the focus of our future research.

## Methods

### Materials

Pharmaceutical grade propylene glycol, glycerol, ethyl acetate, hexane, petroleum ether, ethanol, and KOH were purchased from Fischer Scientific (Thermo Fisher Scientific, Canada). Plant-based CBD isolate material (> 99% purity) was purchased by an independent manufacturer (The Valens Company, Canada), under authorization from Health Canada. Reactions were performed in disposable VWR® 1 dram (4 mL) or 0.5 dram (2 mL) vials. Column chromatography was performed using SiliaFlash F60 (40–63 mm) silica (Silicycle, Canada). Thin-layer chromatography was run on silica gel 60 F_254_ aluminium sheets (Merck, Canada) and visualized by UV fluorescence (254 nm), followed by KMnO_4_. Sun lamp experiments used a 275 W tanning light bulb (General Electric, Canada). White light experiments used a CF1EL/MICRO/865 bulb with a colour temperature of 6500 K (Sylvania, Canada). A Royal Blue Rebel LED on a SinkPAD-II 40 mm round 7-Up base with the power supply controlled by a XANTREX LXQ 30–2 Dual Power Supply was used to supply 448-nm light.

### NMR spectroscopy

^1^H and ^13^C NMR spectra were obtained using a Bruker Avance 300 MHz or Avance 400 MHz spectrometer. ^1^H and ^13^C chemical shifts (δ) are reported in parts per million (ppm) relative to the residual solvent peak (DMSO-*d*_6_: ^1^H, d = 2.50 ppm; ^13^C, d, 39.52 ppm). Coupling constants are reported in hertz (Hz). Splitting patterns are reported as singlet (s), doublet (d), triplet (t), quartet (q), pentet (p), multiplet (m), doublet of doublets (dd), and triplet of doublets (td).

On the Avance-300 spectrometer, heteronuclear single quantum coherence (HSQC) spectra were recorded as a 1496´ 256 matrix with 8 transients per t1 increment and processed as a 2048´ 1024 matrix; the one-bond heteronuclear coupling value was set to 145 Hz. Heteronuclear multi bond correlation (HMBC) spectra were recorded as a 1496´ 256 matrix with 32 transients per t1 increment and processed as a 2048´ 1024 matrix; the long-range coupling value was set to 8 Hz.

On the Avance 400 MHz spectrometer, HSQC spectra were collected as a 1025´ 256 matrix with 16 transients per t1 increment and processed as a 2048´ 1024 matrix; the one-bond heteronuclear coupling value was set to 145 Hz; HMBC spectra were recorded as a 2048´ 256 matrix with 40 transients per t1 increment and processed as a 2048´ 1024 matrix; the long-range coupling value was set to 8 Hz.

### HPLC conditions

5 mg of CBD Isolate (powder) was accurately weighed into amber vials and diluted in acetonitrile to achieve approximately 100 μg/mL of CBD. For e-liquid analysis, 1 g of e-liquid (5% by mass CBD) was weighed into amber vials flask and diluted with acetonitrile to achieve approximately 100 μg/mL of CBD. Individual aliquots from 3 vials for each analysis were then analysed by HPLC/MS/MS.

Samples were transferred to 2 mL LC/MS vials before analysis on an Exion HPLC-SCIEX QTRAP® Triple Quadrupole 5500 + LC–MS/MS System. 1 µL of each sample was injected onto a Raptor ARC-18 column (4.6 mm × 150 mm, 2.7 µm) with a flow rate of 0.5 mL/min, column temperature of 30 °C. Mobile phase A was Milli-Q water + 0.1% formic acid, and mobile phase B was LC/MS grade acetonitrile + 0.1% formic acid. An isocratic elution programme of 75% B for 20 min, increasing to 95% B at 21 min, hold at 95% B for 5 min, decreasing to 75% B at 26.1 min, and lastly hold at 75% B for 4 min was conducted. The total run time was 30 min. A standard solution of 100 ng/mL HU-331 was analyzed individually to determine its retention time and optimise the MRM transitions by direct infusion to MS/MS.

### Mass spectrometry

Detection of HU-331 was using the SCIEX Triple Quad 5500 + mass spectrometer operated in multiple reaction monitoring (MRM) mode. Ionisation was performed using a Turbo V™ ion source using electrospray ionization (ESI) in positive mode. The ion source temperature and the ion spray voltage were set to 550 ^◦^C and 4000 V, respectively. Ion source gas 1 was set to 50 psi and ion source gas 2 was set to 40 psi. Nitrogen was used as the collision gas and the curtain gas, with a flow ratio of 8 psi and 25 psi, respectively.

Low-resolution mass spectra of the HU-331 anion were recorded by using direct infusion mass spectrometry analysis on a Bruker Ultra-High-Resolution Q-Time-Of-Flight Impact II mass spectrometry with an electrospray ionization source. MS data were acquired in full-scan negative ion mode under the following conditions: dry gas temperature, 220 °C; dry gas flow, 7 L/min; nebulizer gas pressure, 1.6 bar; capillary voltage, 3000 V; and spectra rate, 2 Hz. The mass spectrometer was calibrated with sodium formate prior to analysis to ensure mass accuracy (< 10 ppm). High resolution mass spectra were recorded using a Jeol AccuTOF-GCy 4G spectrometer equipped with a field desorption/ionization ion source.

LC/MS (ESI +) data were acquired using the data-independent analysis (DIA) scan mode of a Bruker maXis II QTOF mass spectrometer equipped with an Agilent 1260 HPLC. The scan range was set between 50 and 3000 m/z with precursor ion components automatically selected for fragmentation by collision-induced dissociation (CID, 15–40 V sweep). CID-MSMS spectral data were extracted and aligned using MS-Dial 4.8, after conversion to non-proprietary format (*.ibf). Molecular components of the mixture, found by DIA, were selected based on the following criteria: the precursor ion area is above 10^5^ counts, the product ion fragment signal is over 4000 counts, the retention time is between 0.5 and 25 min, and the product ion signal matches the *m/z* of characteristic fragments (a quinone-like core and terpene-like moiety). The tandem mass spectra of candidate ions were compared against an open-source MSMS spectral library (GNPS) to determine spectral similarity and identify the components with high accuracy.

### Ultraviolet–visible spectroscopy

UV–visible spectra were obtained on a Cary 5000 UV–Vis spectrophotometer using Quartz cells with a 10 mm path length.

### Modelling and calculations

All DFT and TD-DFT calculations were carried out using the ORCA (v. 4.2.1) quantum chemistry suite. Geometries were fully optimized in gas phase without symmetry constraints, employing the B3LYP/ def2-TZVP level of theory. Minima were confirmed using analytical frequency calculations. TD-DFT spin unrestricted calculations were performed using B3LYP/ def2-TZVP including relativistic effects (ZORA) and employing the RIJCOSX keyword; 50 roots were included in the TD-DFT calculation space. Molecular orbitals, spin densities, and spin density transition differences were visualized and plotted using Avogadro and Chemcraft.

## Supplementary Information

Supplementary Information.

## Data Availability

All data generated or analysed during this study are included in this published article (and its Supplementary Information file).
