# Supplementary material for: CBD hydroxyquinone photo-isomerises to a highly reactive intermediate
Source: Sci Rep. 2023 Apr 28;13:6967. doi: 10.1038/s41598-023-33815-7 (PMC10147596; doi:10.1038/s41598-023-33815-7)
Supplement: Supplementary file 1 — Supplementary Information. [file 41598_2023_33815_MOESM1_ESM.docx]

**Supporting Information**

**CBD Hydroxyquinone Photo-Isomerises to a Highly Reactive
Intermediate**

Brodie. J. Thomson^1^, Summer Hanna^2^, Adrian Schwarzenberg^2^, Pirouz Kiani^3^, Dan Bizzotto^1^, Pierre Kennepohl^3^, Ashley Davies^2^*, Markus Roggen^4^*, Glenn M. Sammis^1^*

^1^Department of Chemistry, University of British Columbia; Vancouver, Canada

^2^Group Research and Development Centre, BAT Investments Limited; Southampton, United Kingdom

^3^Department of Chemistry, University of Calgary; Calgary, Canada

^4^DELIC Labs; Vancouver, Canada

*Corresponding authors: ashley_davies1@bat.com; markus@deliclabs.com; gsammis@chem.ubc.ca

## HU-331 Content in CBD E-liquids

**Figure S1.** Observed colour change of fresh CBD e-liquid solutions aged in a) the dark and b) natural light/dark cycles for 10 weeks under ambient laboratory conditions (~25 °C, ~60% relative humidity). [HU-331] denotes concentration of HU-331.

a) 10 weeks dark storage


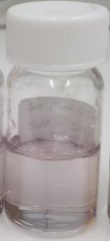


b) 10 weeks light/dark storage


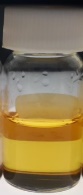


[HU-331] = 116.03 μg/g

[HU-331] =25.04 μg/g


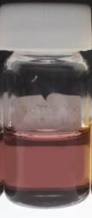


5% CBD e-liquid

[HU-331] = 0.05 μg/g

## Synthesis of HU-331 (**2**)

HU-331 was synthesized and isolated according to the procedure previously reported by Mechoulam and coworkers.^1^

**^1^H NMR (300 MHz, DMSO-*d*_6_)** δ 10.38 (s, 1H), 6.42 (s, 1H), 5.09 (s, 1H), 4.50 (s, 1H), 4.45 (s, 1H), 3.67 – 3.55 (m, 1H), 2.76 (td, *J* = 12.2, 2.8 Hz, 2H), 2.31 (td, *J* = 7.5, 1.45 Hz, 1H), 2.16 – 2.03 (m, 1H), 2.0 – 1.89 (m, 1H), 1.72 – 1.64 (m, 1H), 1.60 (s, 4H), 1.55 (s, 3H), 1.50 – 1.38 (m, 2H), 1.33 – 1.21 (m, 4H), 0.86 (t, *J* = 6.91 Hz, 3H).

**^13^C{H} NMR (75 MHz, DMSO-*d*_6_)** δ 187.8, 184.3, 154.1, 148.8, 145.3, 133.9, 132.5, 124.2, 122.5, 110.9, 44.3, 35.7, 31.4, 30.7, 29.1, 28.2, 27.4, 23.8, 22.4, 19.2, 14.4.

## Isolation of HU-331 anion (**3**)

To an open 20 mL vial was dissolved CBD (150 mg, 0.48 mmol) in hexane (10 mL). Crushed KOH (200 mg, 3.56 mmol) was charged to the vial and stirred for 5 hours. The precipitate was filtered, washed with hexane and ethyl acetate:hexane (20:80), and dissolved in ethyl acetate before the solvent was removed under reduced pressure to afford a dark purple oil. The oil was recrystallized in ethyl acetate:hexane to afford wet purple solid. Removal of solvent resulted in decomposition of the isolate from purple to orange.

**^1^H NMR** **(300 MHz, DMSO-*d*_6_)** δ 6.0 (s, 1H), 5.03 (s, 1H), 4.44 (s, 1H), 4.37 (s, 1H), 3.59 – 3.50 (m, 1H), 3.0 (td, *J* = 12.5, 2.2 Hz, 1H), 2.1 (t, *J* = 7.3 Hz, 1H), 1.89 – 1.78 (m, 1H), 1.66 – 1.56 (m, 2H), 1.54 (s, 6H), 1.41 – 1.30 (m, 2H), 1.30 – 1.20 (m, 4H), 0.85 (t, *J* = 6.8 Hz, 3H).

**^13^C{^1^H} NMR (75 MHz, DMSO-*d*_6_)** δ 188.9, 181.5, 169.3, 150.2, 140.5, 138.2, 129.8, 128.0, 115.1, 108.7, 43.2, 35.2, 31.1, 30.4, 29.8, 28.0, 27.3, 23.4, 21.9, 19.5, 13.9.

**HRMS** (ESI) *(m/z)* calculated for C_21_H_27_O_3_^-^ [M] 327.1966, found 327.1920.

### Supporting NMR spectroscopic data

**Figure S2.** HU-331 (**2**) ^1^H NMR (300 MHz, DMSO-­*d_6_*)
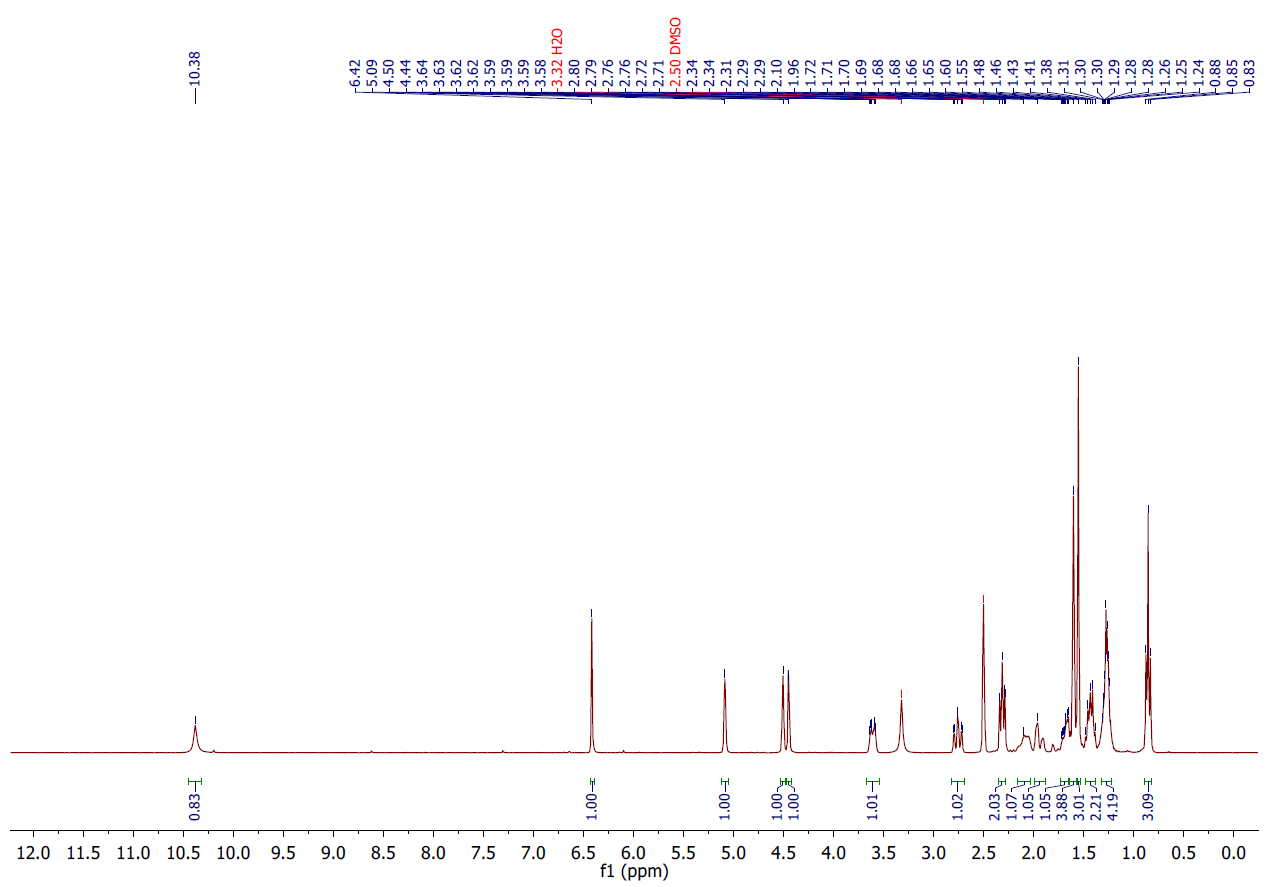


**Figure S3.**
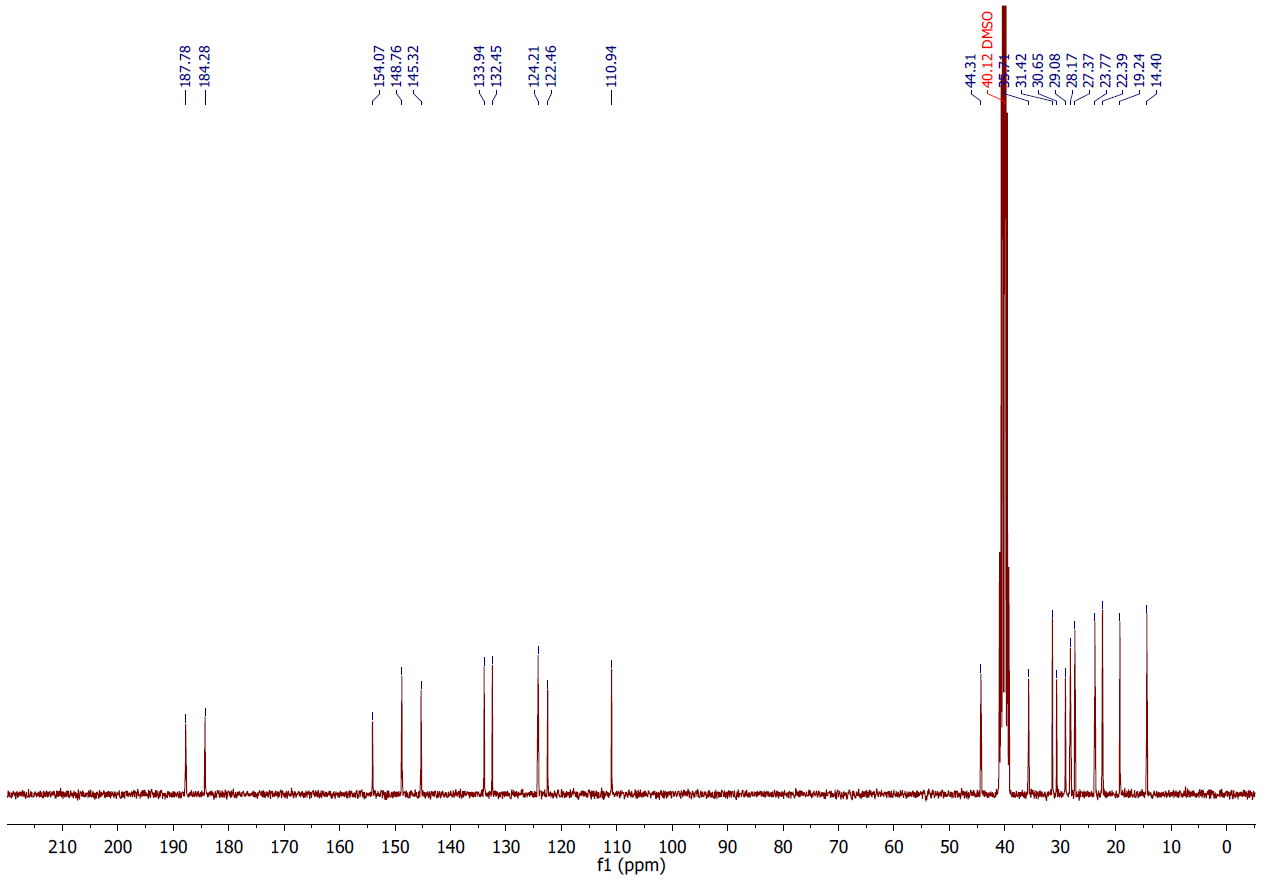
HU-331 (**2**) ^13^C{^1^H} NMR (300 MHz, DMSO-­*d_6_*)

**Figure S4.** HU-331 anion (**3**) ^1^H NMR (300 MHz, DMSO-­*d_6_*)
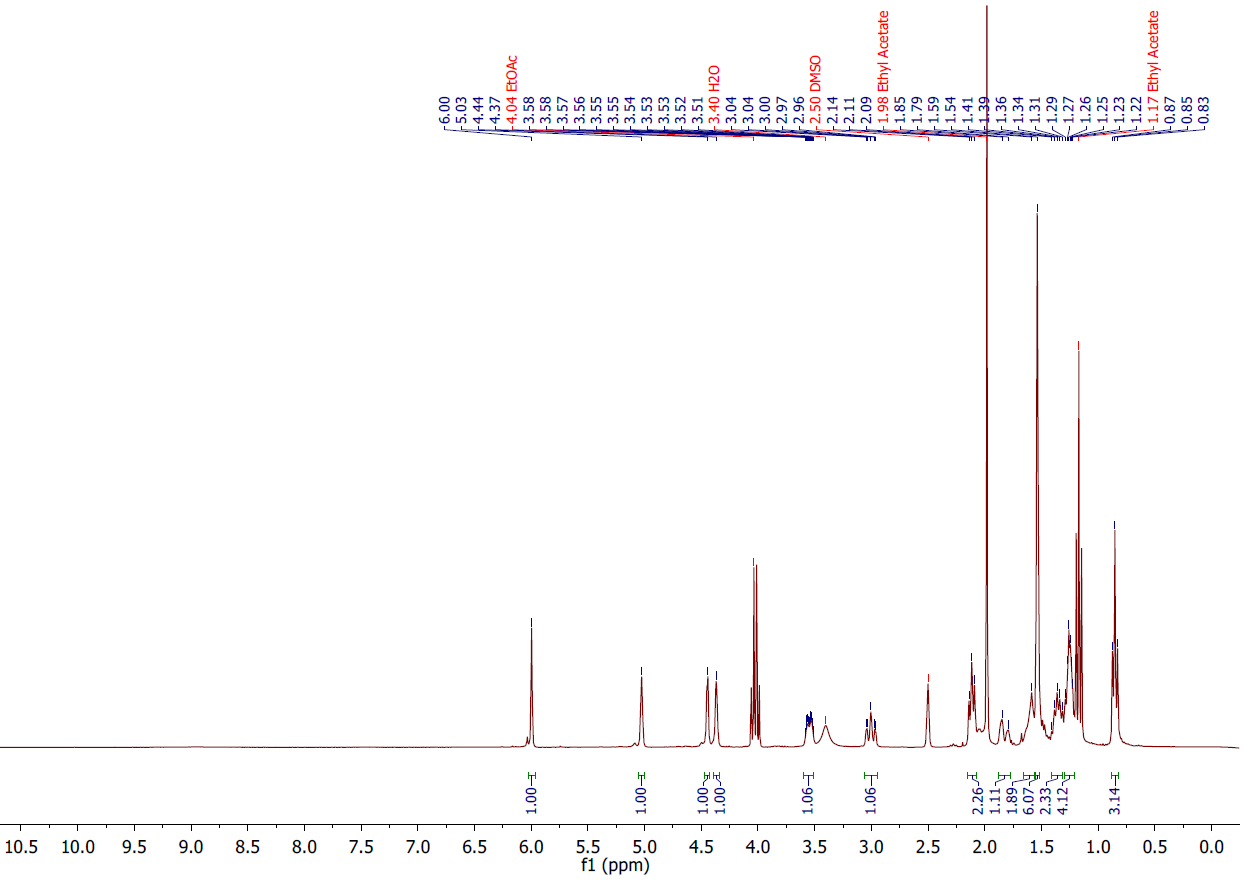


**Figure S5.**
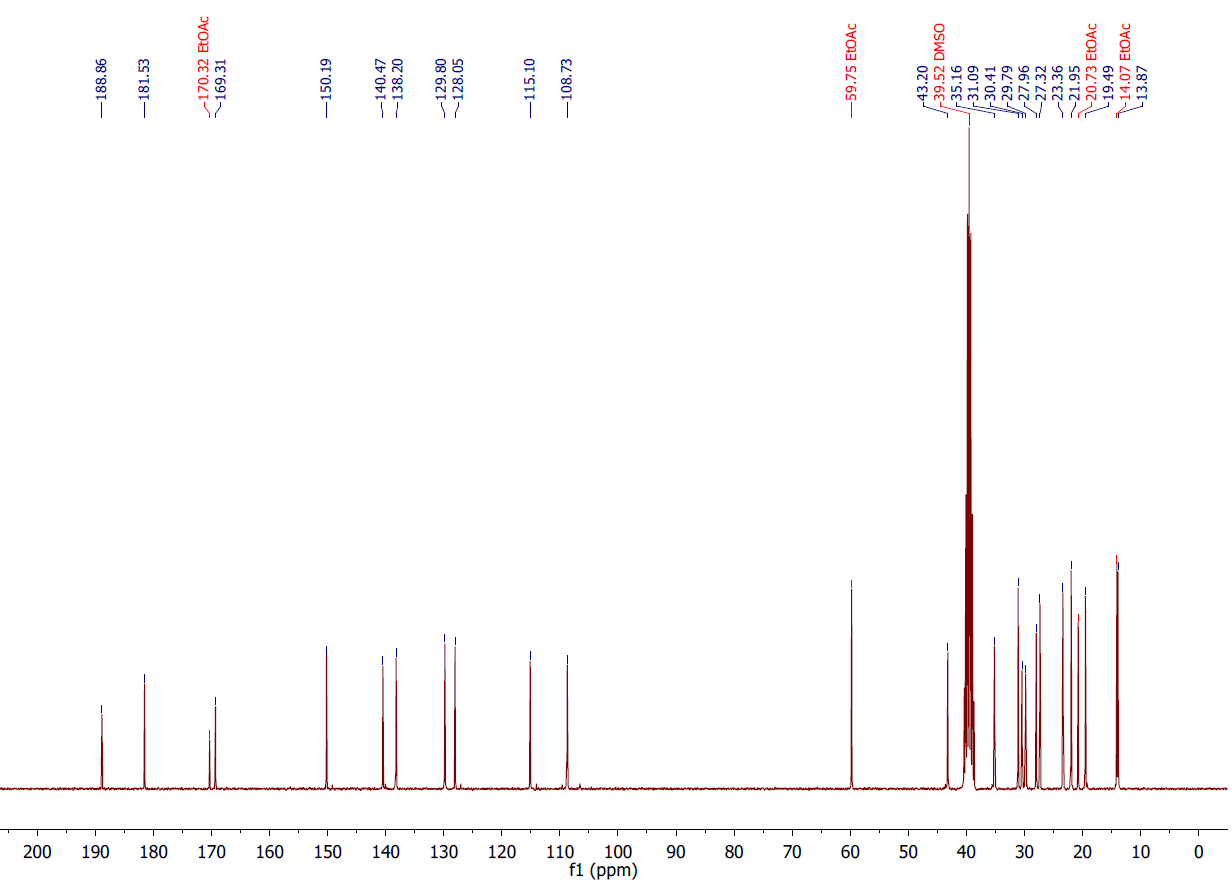
HU-331 anion (**3**) ^13^C{^1^H} NMR (300 MHz, DMSO-­*d_6_*)

**Figure S6.** HU-331 anion (**3**) HSQC (300 MHz, DMSO-*d*_6_)
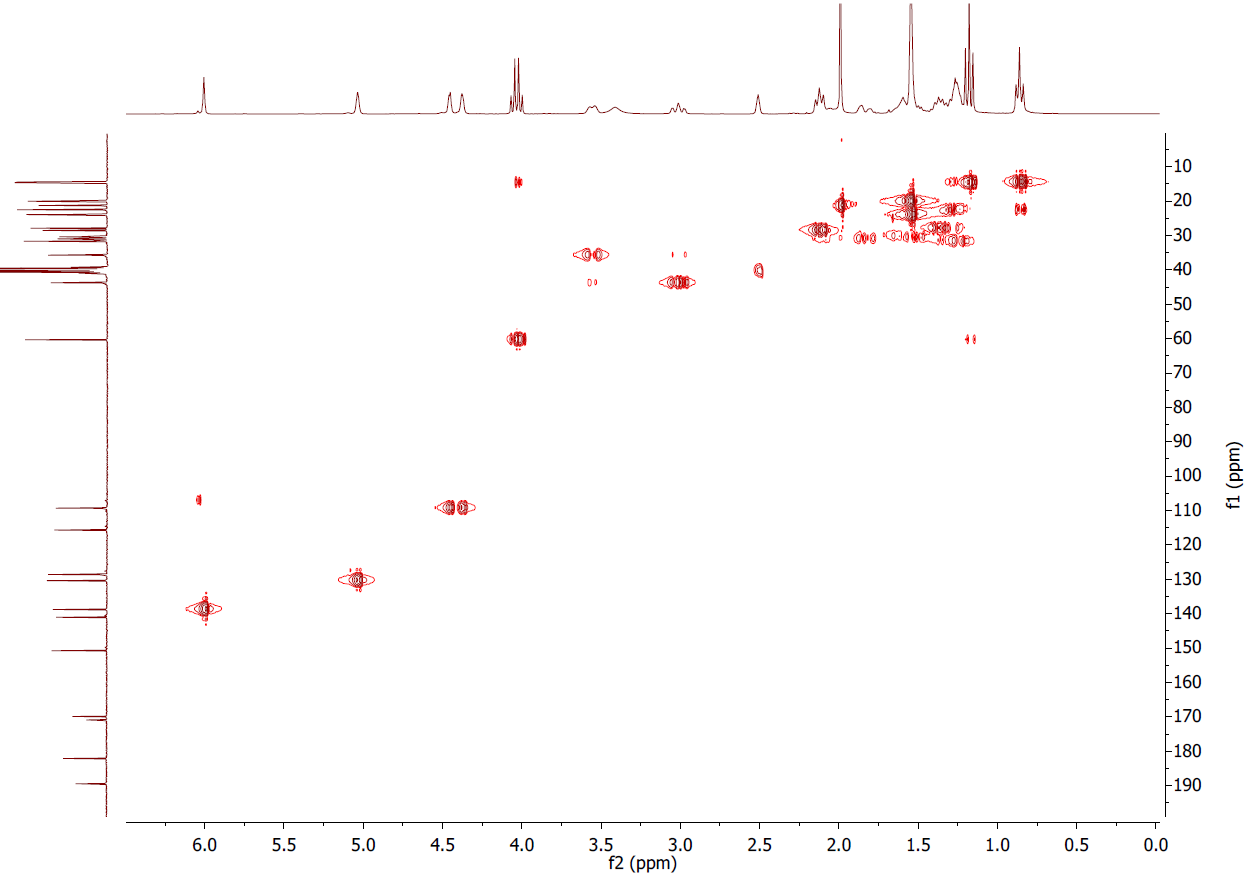


**Figure S7.**
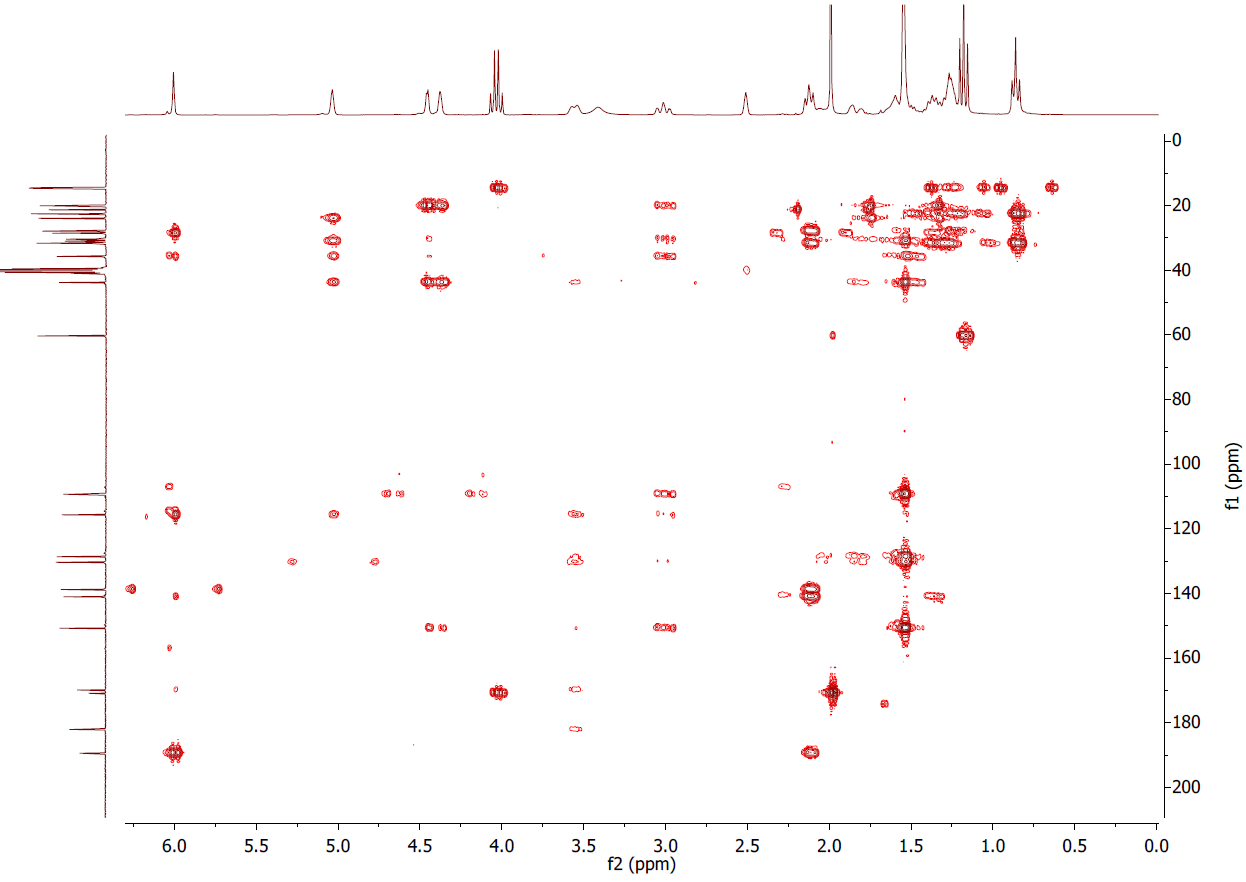
HU-331 anion (**3**) HMBC (300 MHz, DMSO-*d*_6_)

## HU-331 (**2**) degradation by white light

1,4-dinitrobenzene solution in DMSO-*d_6_* (0.25 mL, 0.03 M) and HU-331 (10 mg, 0.03 mmol) were dissolved in DMSO-*d_6_* (4.75 mL), wrapped in aluminium foil, and immediately analysed by ^1^H NMR to identify the initial HU-331:1,4-dinitrobenzene area ratio. 0.5 mL aliquots were added to NMR tubes and placed 6 cm from a white light source for times of 10, 20, 30, 40, 50 and 60 minutes before being analysed by ^1^H NMR spectroscopy. A subsequent 0.5 mL aliquot was stored in the dark for 60 minutes before being analysed again by ^1^H NMR spectroscopy.

Using the formula below, HU-331 degradation was determined relative to the starting concentration:

$$\left( \frac{Integradation of HU331 relative to dinitrobenzne at T_{x}}{Inegrated area of HU331 relative to dinitrobenzene at T_{0}} \right)$$

### Supporting NMR spectroscopic data

**Figure S8.** HU-331 (**2**) T_0_ ^1^H NMR (300 MHz, DMSO-*d*_6_)

**
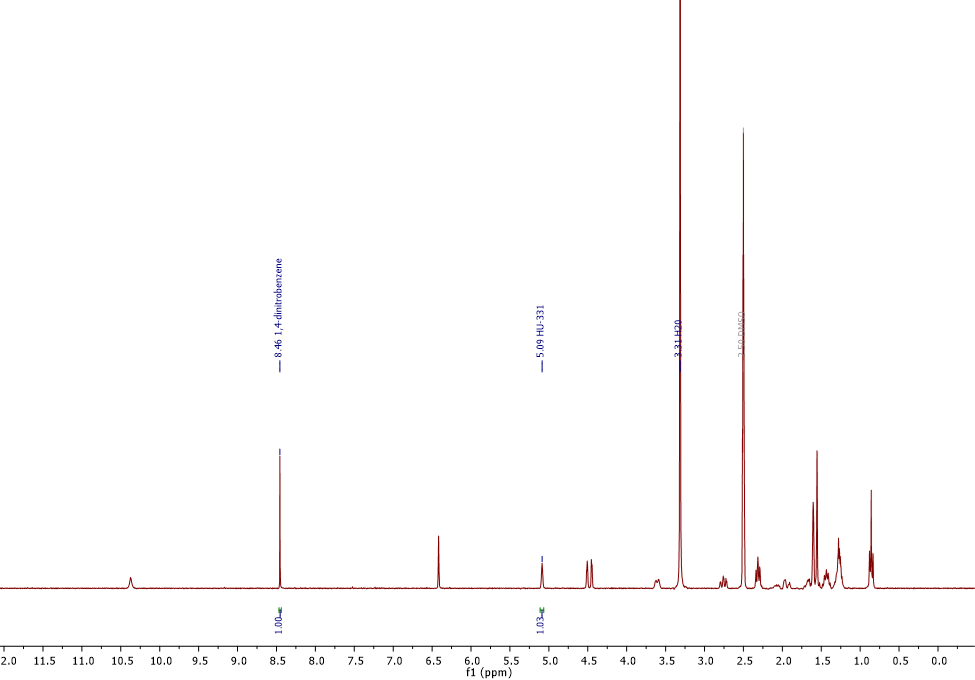
**

**Figure S9.** HU-331 (**2**) 10 min Light ^1^H NMR (300 MHz, DMSO-*d*_6_)

**Figure S10.** HU-331 (**2**) 20 min Light ^1^H NMR (300 MHz, DMSO-*d*_6_)

**Figure S11.** HU-331 (**2**) 30 min Light ^1^H NMR (300 MHz, DMSO-*d*_6_)

**Figure S12.** HU-331 (**2**) 40 min Light ^1^H NMR (300 MHz, DMSO-*d*_6_)

**Figure S13**. HU-331 (**2**) 50 min Light ^1^H NMR (300 MHz, DMSO-*d*_6_)

**Figure S14.** HU-331 (**2**) 60 min Light ^1^H NMR (300 MHz, DMSO-*d*_6_)

**Figure S15.** HU-331 (**2**) 60 min Dark ^1^H NMR (300 MHz, DMSO-*d*_6_)

### TableS1: ^1^H NMR areas of HU-331 relative to the dinitrobenzene internal standard

**Table S1.** ^1^H NMR areas of HU-331 (**2**) relative to the dinitrobenzene internal standard. ^a^ Ratio between HU-331 (5.09 ppm) and the hydroxyquinol **5** (5.40 ppm) was used to determine the relative concentration.

|  | Run 1 | | Run 2 | | Run 3 | |
| --- | --- | --- | --- | --- | --- | --- |
| Time in light | Relative to Std. | Relative to T_0_ | Relative to Std. | Relative to T_0_ | Relative to Std. | Relative to T_0_ |
| 0 | 1.06 | 1 | 1.1 | 1 | 1.03 | 1 |
| 10 | 0.88 | 0.83 | 0.91 | 0.83 | 0.86 | 0.83 |
| 20 | - | 0.7^a^ | 0.76 | 0.69 | 0.70 | 0.68 |
| 30 | 0.62 | 0.58 | 0.63 | 0.57 | 0.57 | 0.55 |
| 40 | 0.42 | 0.40 | 0.53 | 0.48 | 0.50 | 0.49 |
| 50 | 0.4 | 0.38 | 0.41 | 0.37 | 0.39 | 0.38 |
| 60 | 0.32 | 0.30 | 0.32 | 0.29 | 0.31 | 0.30 |
| 60 (Dark) | 1.06 | 1 | 1.1 | 1 | 1.03 | 1 |

## Visible light absorption comparison between CBD and HU-331

**Figure S16:** Visible light absorbance spectra for HU-331 and CBD at 0.5 mg mL^-1^. Λ_max_ of HU-331, 413 nm. No absorption in the visible range observed for CBD.

## LED general experimental setup

Vials were placed 5 cm from an LED light source with a wavelength of 448 nm. Vials were marked in place to maintain positions between experiments and placed within the central focus of the light for 15 minutes. The power to the light source was limited to 20 V and 0.5 A, and was covered for the duration of the experiment.


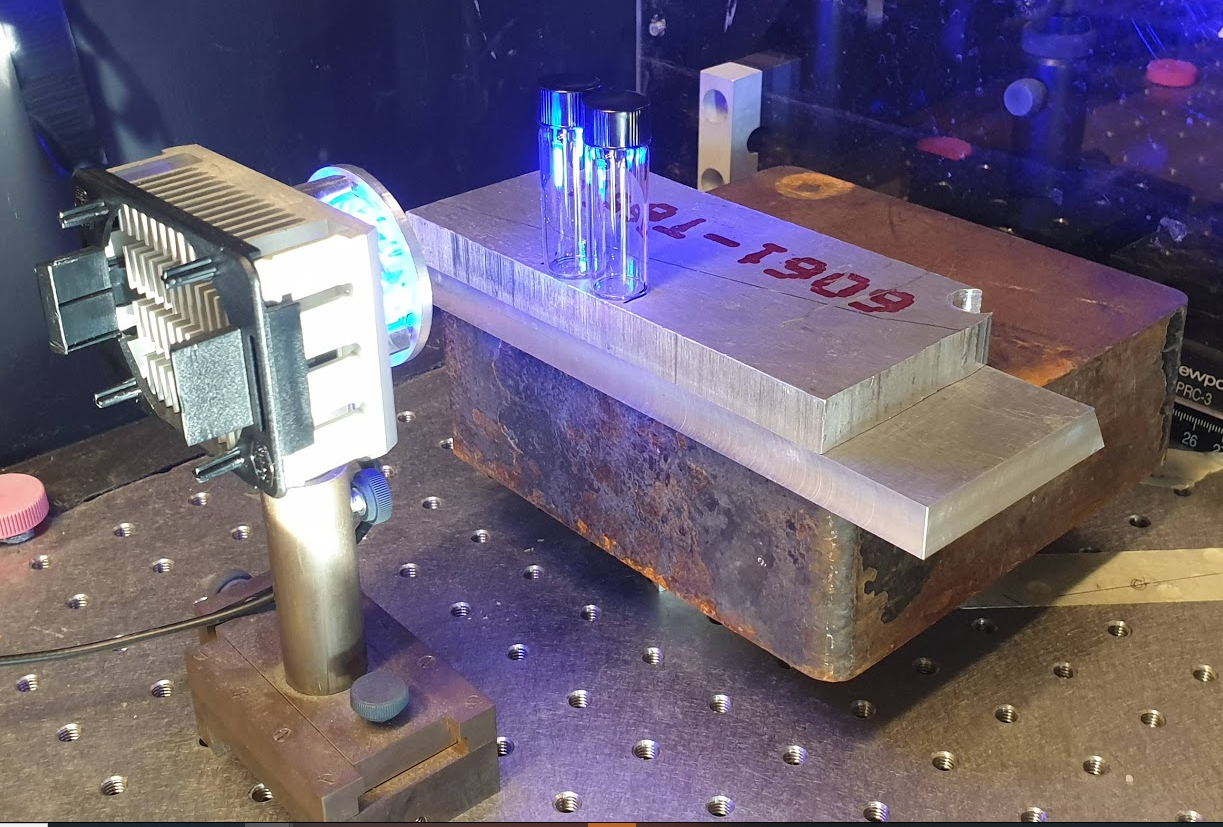


**Figure S17.** Experimental Setup for LED Irradiation

## Comparison of CBD (**1**) and HU-331 (**2**) exposure to 448 nm of Light

To a 2 mL vial was dissolved CBD (**1**) (6 mg, 0.02 mmol) or HU-331 (**2**) (6 mg, 0.02 mmol) in DMSO-*d*_6_ (0.6 mL). Both vials were subject to 448 nm of light described by general experimental setup for 15 minutes before being analyzed by ^1^H NMR spectroscopy.

### Supporting NMR spectroscopic data

**Figure S18.** CBD (**1**) after 15 minutes of light exposure. ^1^H NMR (300 MHz, DMSO-*d*_6_)

**
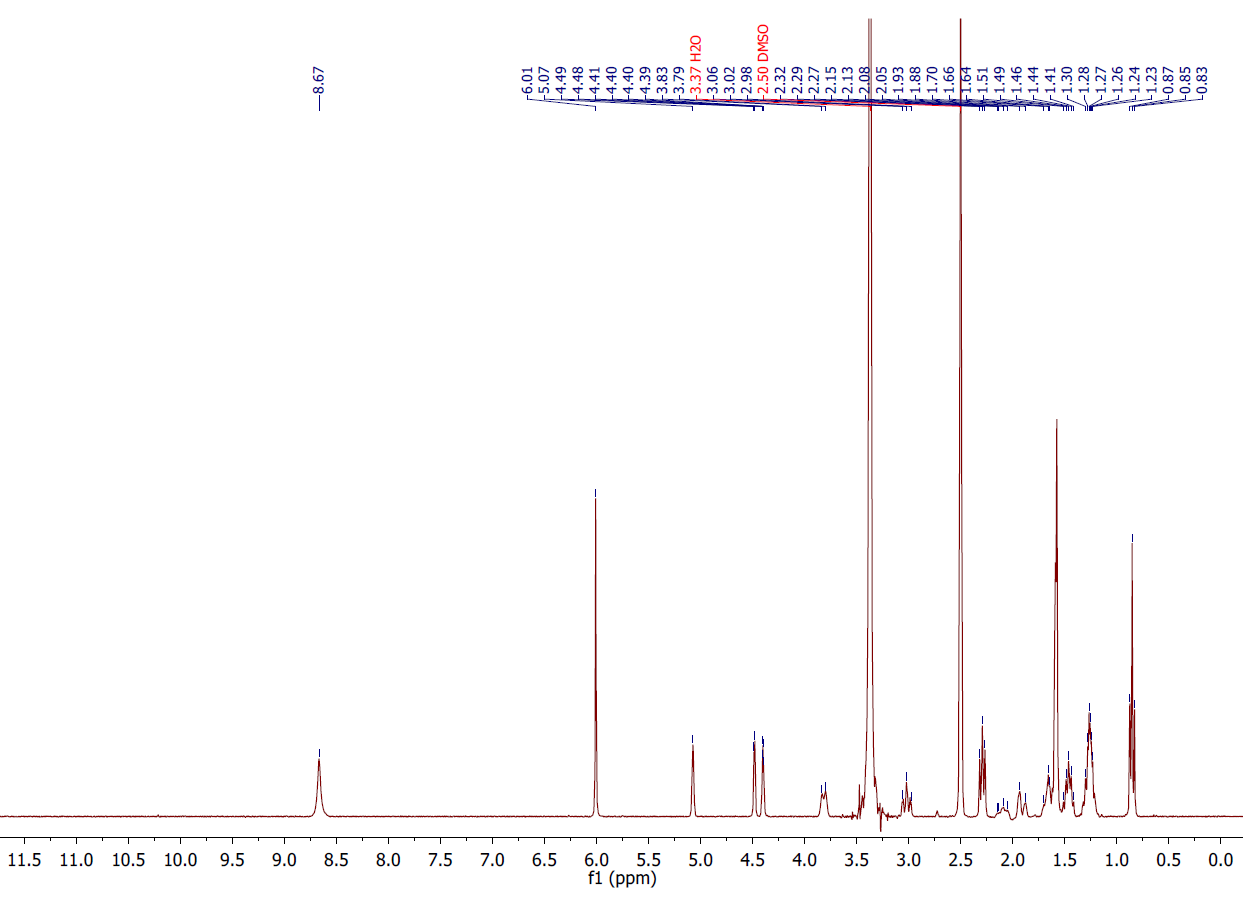
**

**Figure S19.** HU-331 (**2**) after 15 minutes of light exposure. ^1^H NMR (300 MHz, DMSO-*d*_6_)
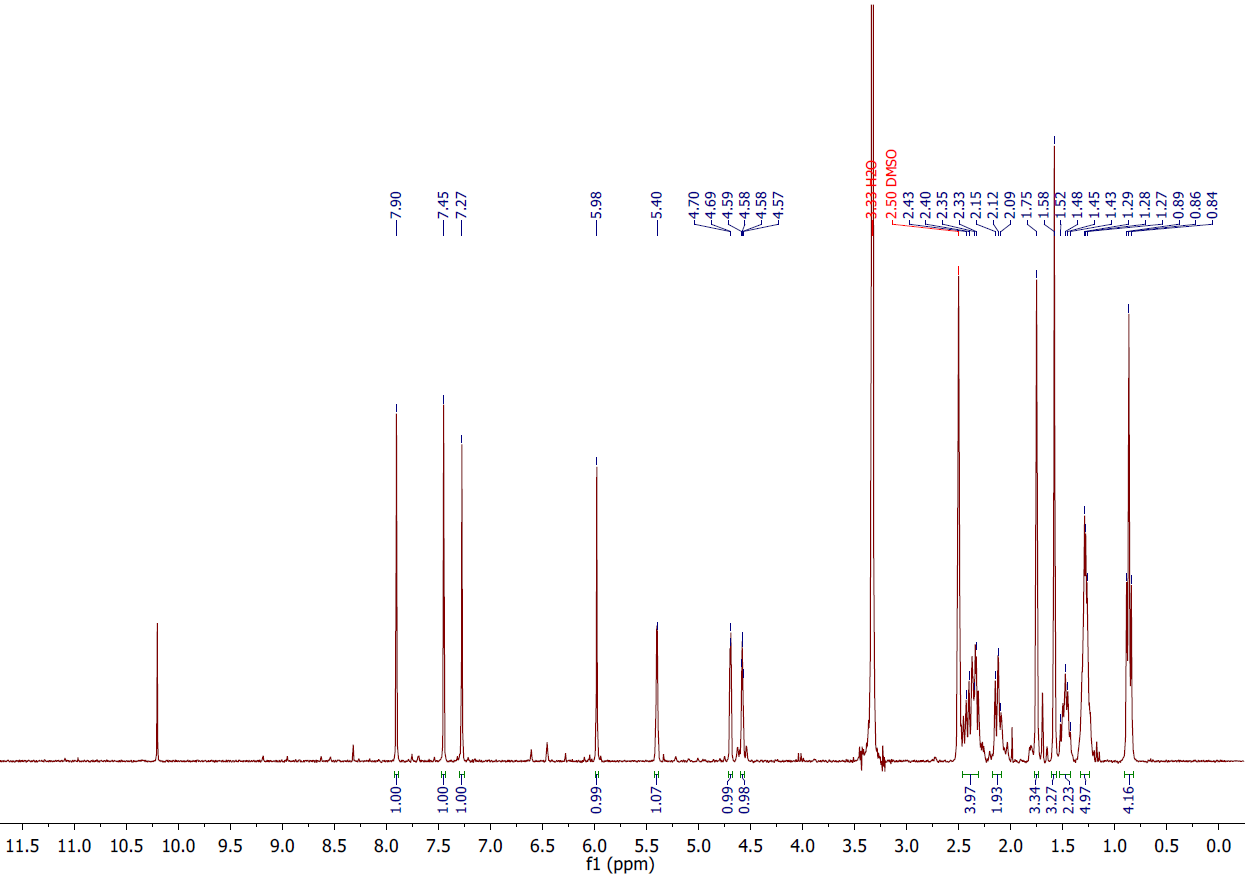


## Influence of CBD (**1**) concentration on the formation of hydroxyquinol **5**

To a 2 mL vial was dissolved CBD (100 mg 0.3 mmol) and HU-331 (1 mg, 0.003 mmol) in DMSO-*d*_6_ (0.6 mL). To a separate 2 mL vial was dissolved CBD (6 mg, 0.02 mmol) and HU-331 (6 mg, 0.02 mmol) in DMSO-*d*_6_ (0.6 mL). Both vials were subject to 448 nm of light described by the general experimental setup for 15 minutes before being analyzed by ^1^H NMR spectroscopy.

### Supporting NMR spectroscopic data

**Figure S20.** [CBD (**1**, red):HU-331 (**2**, blue)] (100:1) After 15 minutes of 448 nm light exposure. ^1^H NMR (300 MHz, DMSO-*d*_6_)


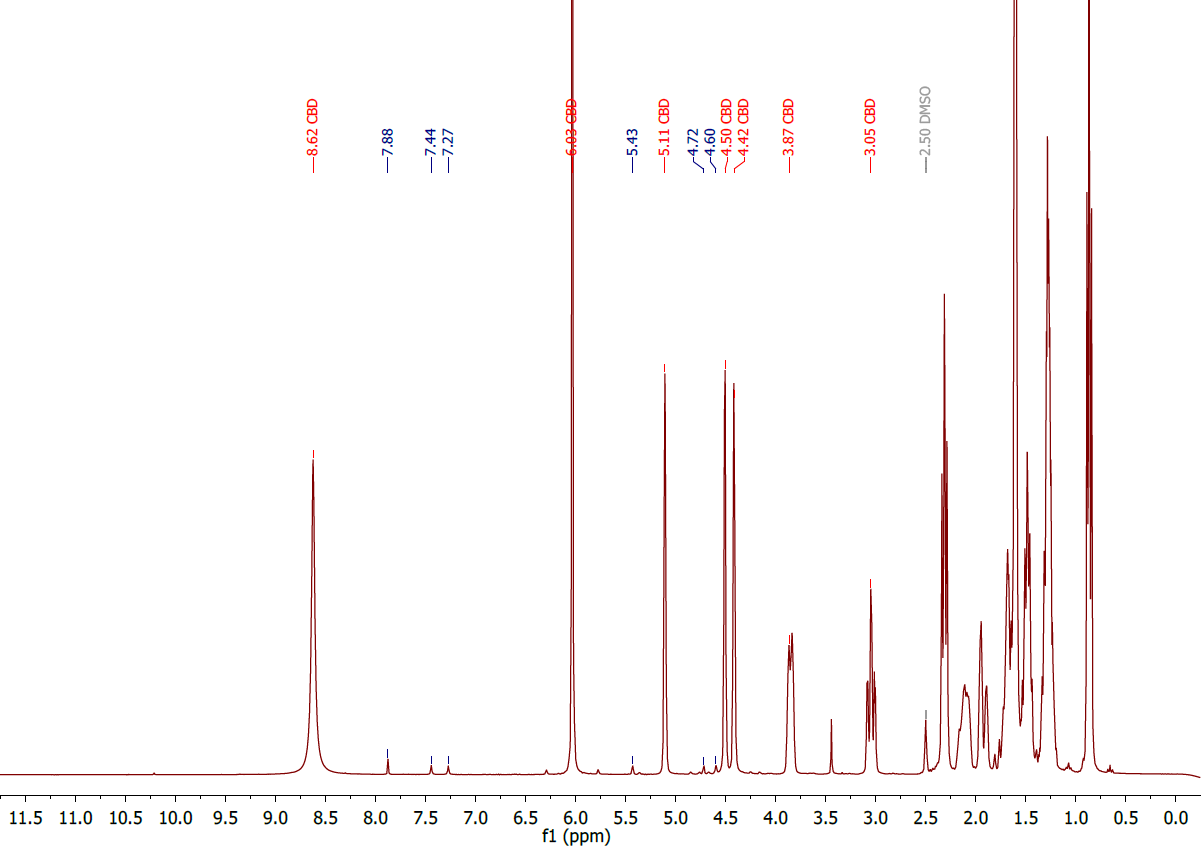


**Figure S21.** [CBD (**1**, red):HU-331 (**2**, blue)] (1:1) after 15 minutes of 448 nm light exposure. ^1^H NMR (300 MHz, DMSO-*d*_6_)
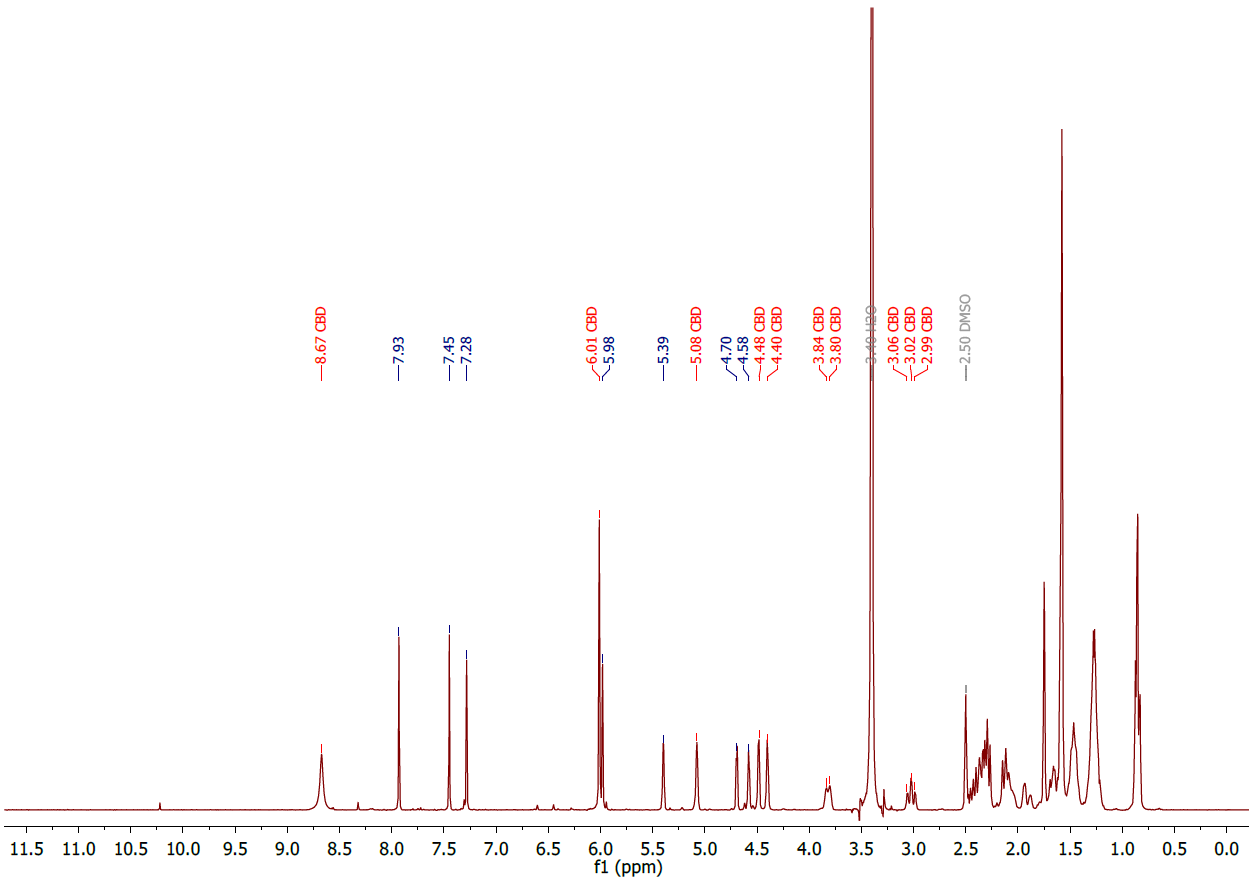


## Formation of hydroxyquinol **5** in solutions of IPA and PG

To a 2 mL vial was dissolved HU-331 (**2**) (2 mg, 0.006 mmol) in solutions of IPA or PG (0.6 mL). The vials were subject to 448 nm of light described in the general experimental setup for 15 minutes. The solutions were immediately analysed by ^1^HNMR spectroscopy.

1^11^

### Supporting NMR spectroscopic data

**Figure S22.** HU-331 (**2**) after 15 minutes of 448 nm light exposure in PG. ^1^H NMR (300 MHz, DMSO-*d*_6_)


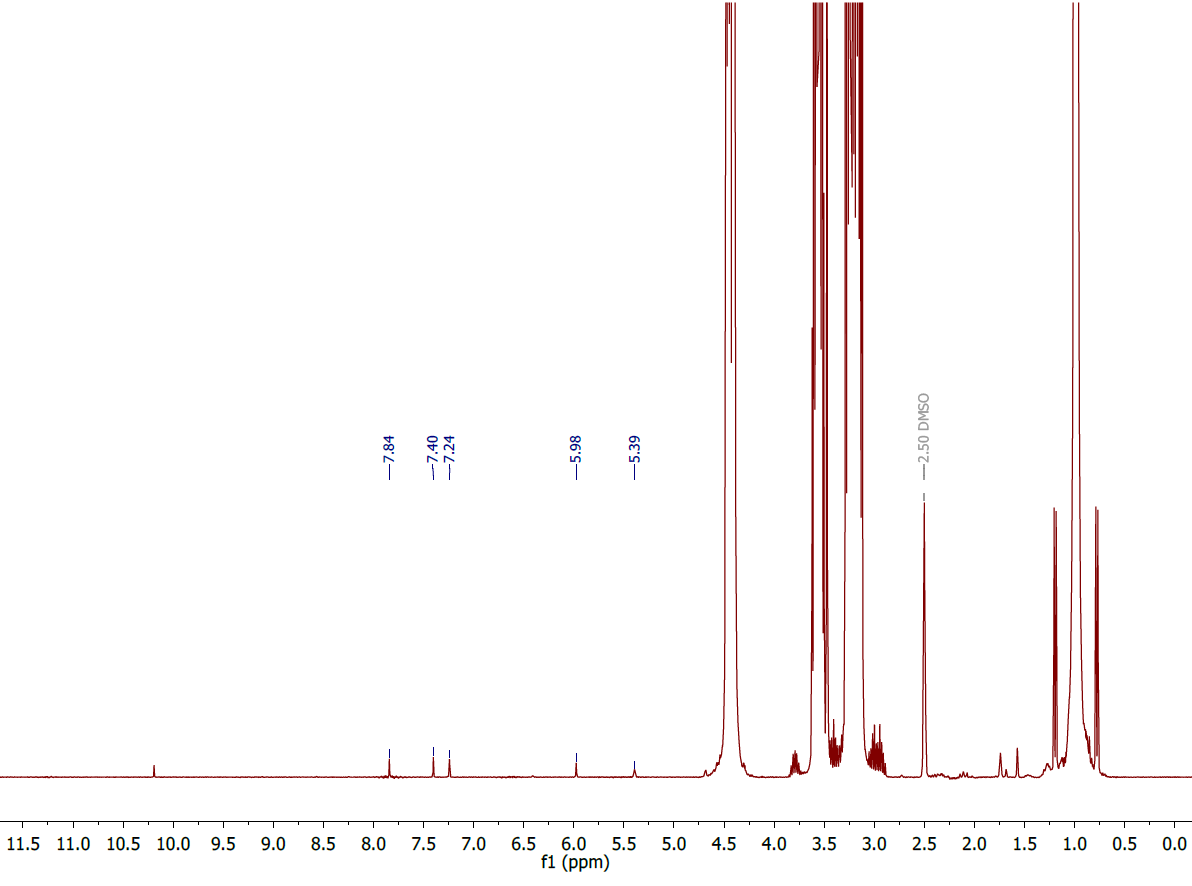


**Figure S23.** HU-331 after 15 minutes of 448 nm light exposure in IPA. ^1^H NMR (300 MHz, DMSO-*d*_6_)
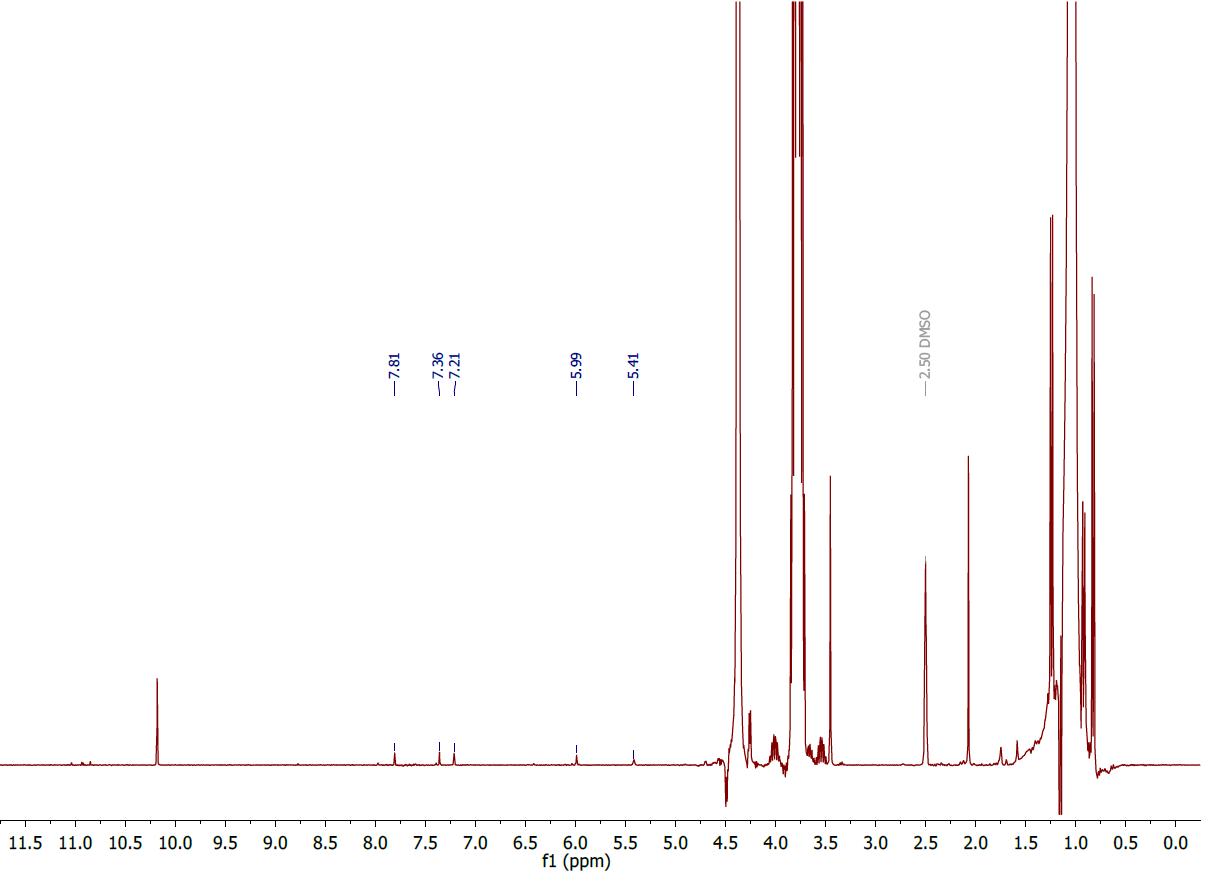


## Influence of oxygen on the stability of hydroxyquinol **5**

*Open reaction conditions*

To a 2 mL vial was dissolved HU-331 (**2**) (6 mg, 0.02 mmol) in DMSO-*d*_6_ (0.6 mL). In a separate 2 mL vial was dissolved HU-331 (**2**) (6 mg 0.02 mmol) and CBD (5.5 mg 0.02 mmol) in DMSO-*d*_6_ (0.6 mL)_._ Both were subject to 448 nm of light for 15 minutes using the general experimental set up before being left for 48 hours and analyzed by ^1^H NMR spectroscopy.

*Oxygen-free conditions*

To a J-Young tube was dissolved CBD (**1**) (6 mg, 0.02 mmol) and HU-331 (**2**) (2 mg, 0.006 mmol) in degassed DMSO-*d*_6_ (0.6 mL). The J-Young was cycled with nitrogen 3 times before being subject to 448 nm of light using the general experimental setup. After 96 hours the solution was analysed by ^1^H NMR spectroscopy.

### Supporting NMR spectroscopic data


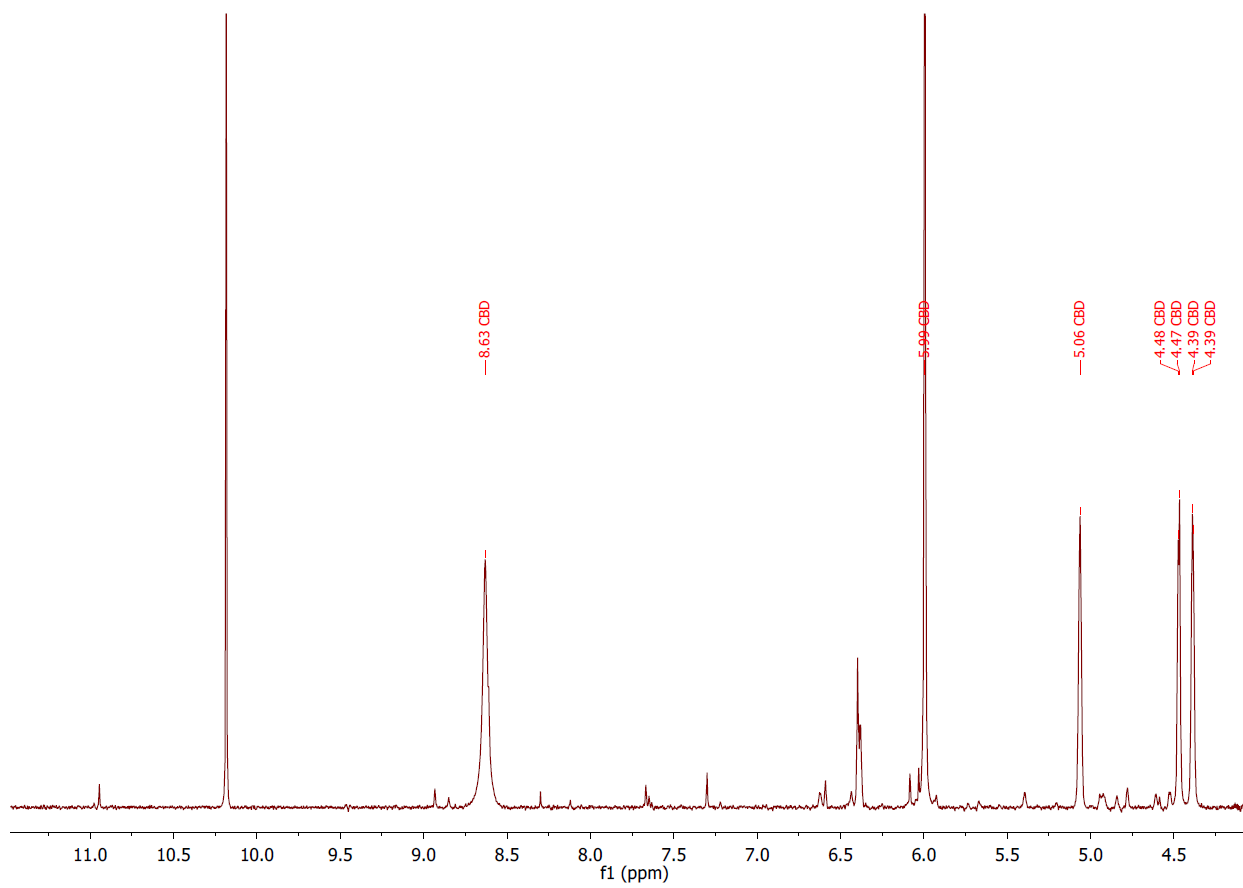
**Figure S24.** CBD (**1**) + HU-331 (**2**) 48 hours after 15 minute exposure to 448 nm light. ^1^H NMR (300 MHz, DMSO-*d*_6_)

**Figure S25.** HU-331 (**2**) 48 hours after 15 minutes exposure to 448 nm light. ^1^H NMR (300 MHz, DMSO-*d*_6_)


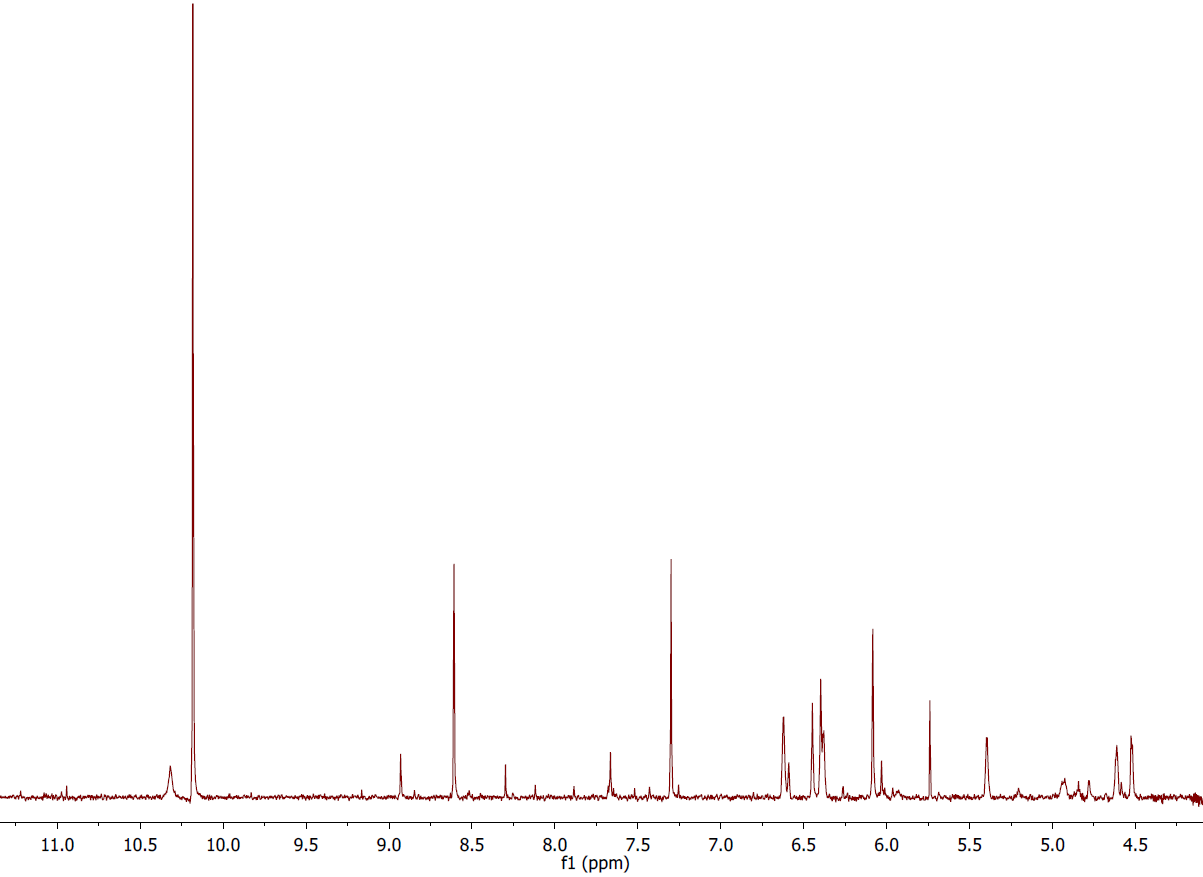


**Figure S26.** Oxygen-free CBD (**1**, red) + HU-331 (**2**, blue) after 15 minutes exposure to 448 nm light. ^1^H NMR (300 MHz, DMSO-*d*_6_)


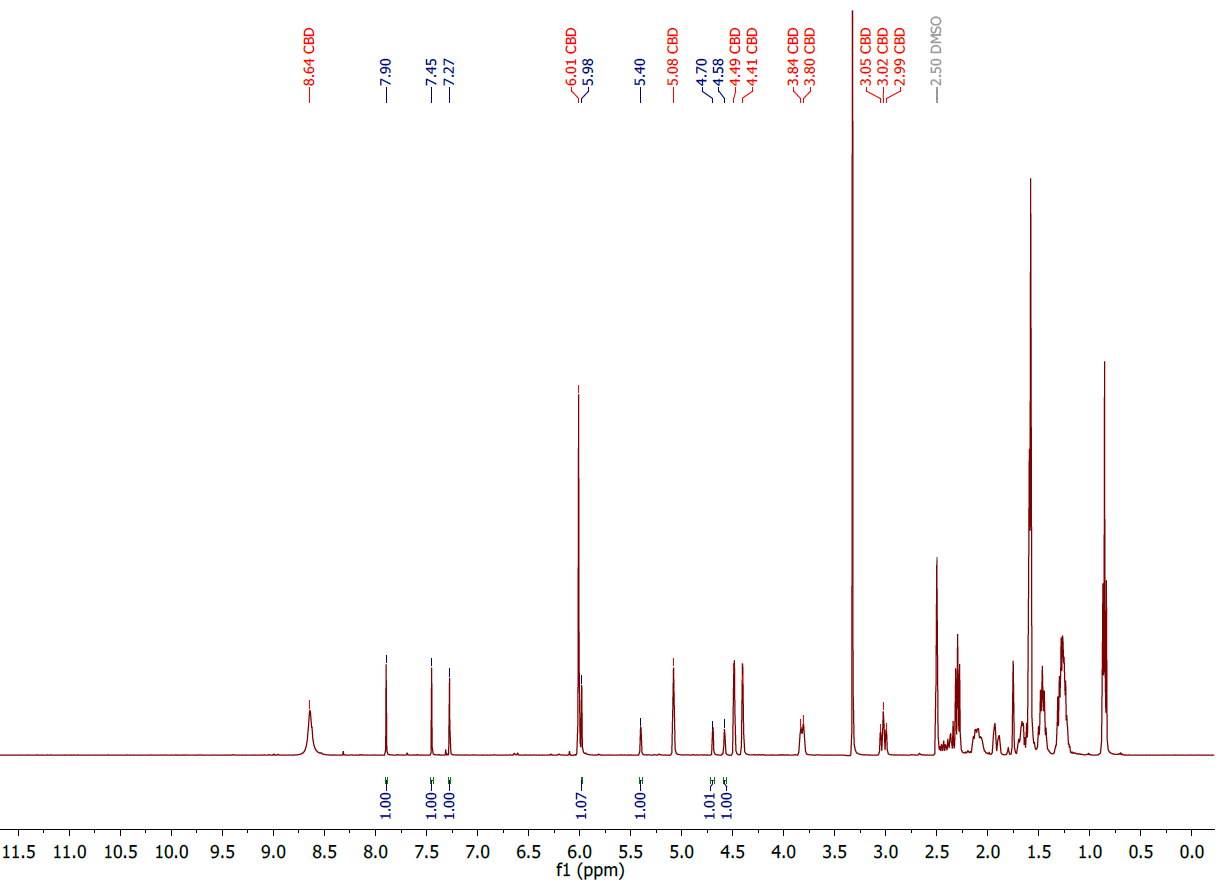


## Identification of labile protons in hydroxyquinol **5**

To a 1 mL vial was dissolved HU-331 (**2**) (2 mg, 0.006 mmol) in DMSO-*d*_6_ before being subject to 448 nm of light described by the general experimental setup for 15 minutes. The solution was analysed by ^1^HNMR spectroscopy before being spiked with D_2_O and re-analyzed.

### Supporting NMR spectroscopic data

**Figure S27.** D_2_O spike for determination of labile protons in hydroxyquinol **5**. HU-331 (**2**) after 15 minutes exposure to 448 nm light (top). Addition of D_2_O (bottom). ^1^H NMR (300 MHz, DMSO-*d*_6_)


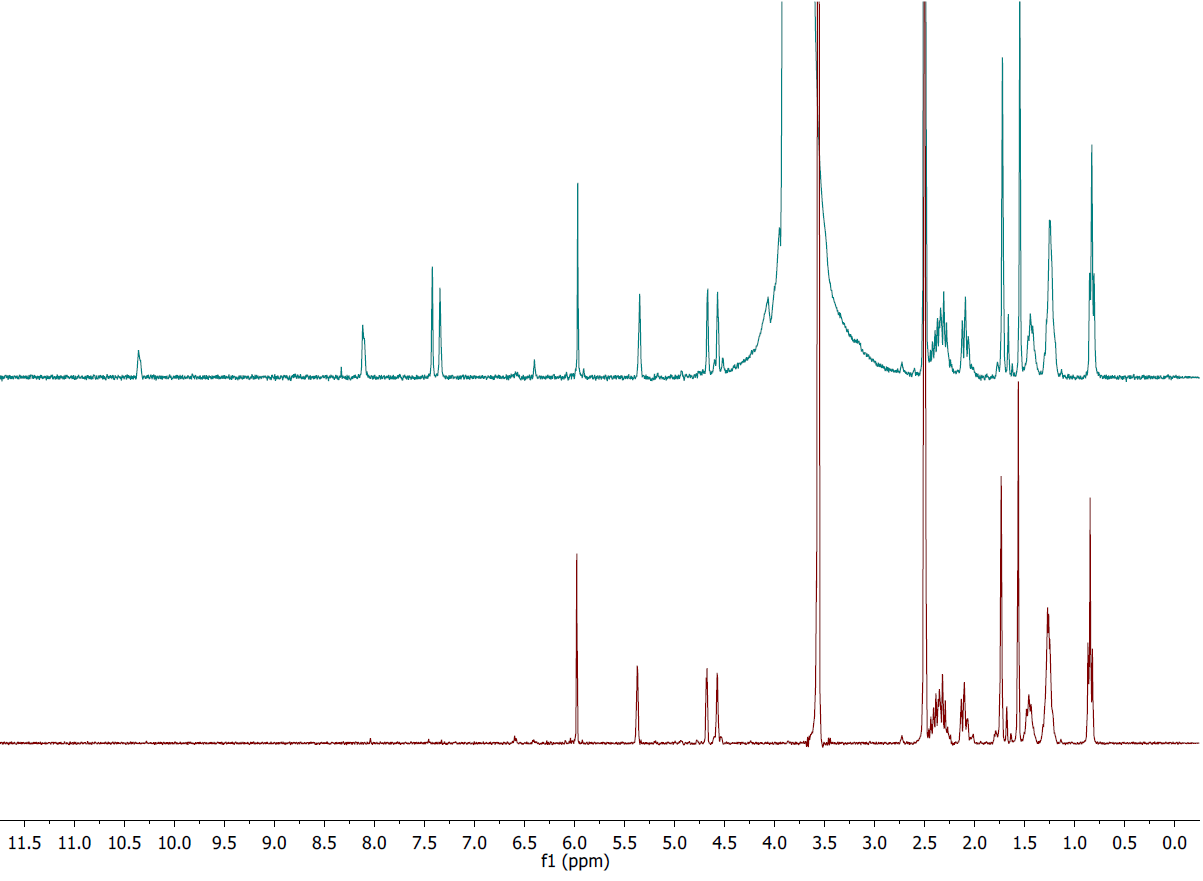


## Isolation of 5’-methyl-4-pentyl-2’-(prop-1-en-2-yl)-3’,4’-dihydro-[1,1’-biphenyl]-2,3,6-triol (**5**)

To a J-Young tube was dissolved HU-331 (**2**) (15 mg) in degassed DMSO-*d*_6_ (0.6 mL). The J-Young was cycled with nitrogen 3 times before being subject to 448 nm of light using the described general experimental set up. The solution was analyzed by ^1^H NMR, ^13^C NMR, HSQC and HMBC spectroscopy indicating a quantitative conversion to product.

**^1^H NMR (400 MHz, DMSO-*d*_6_)** δ 7.90 (s, OH), 7.45 (s, OH), 7.27 (s, OH) 5.99 (s, 1H), 5.41 – 5.39 (m, 1H), 4.71 – 4.68 (m, 1H), 4.59 – 4.57 (m ,1H), 2.48 – 2.27 (m, 4H), 2.16 – 2.08 (m, 2H), 1.75 (s, 3H), 1.58 (s, 3H), 1.52 – 1.42 (m, 2H), 1.34 – 1.22 (m, 4H), 0.86 (t, *J* = 6.9 Hz, 3H).

**^13^C{^1^H} NMR (400 MHz, DMSO-*d*_6_)** δ 147.7, 145.2 (8), 143.8, 134.4, 132.9, 132.5, 128.3, 125.8, 125.0, 115.2, 112.3, 105.6, 31.3, 29.7, 29.2, 28.2, 27.3, 22.7, 21.4, 14.0.

**HRMS** (FD+) (*m/z*) calculated for C_21_H_28_O_3_ [M] 328.2038, found 328.2046.

### Supporting NMR spectroscopic data

**Figure 28.** Hydroxyquinol **5** ^1^H NMR (400 MHz, DMSO-*d*_6_)
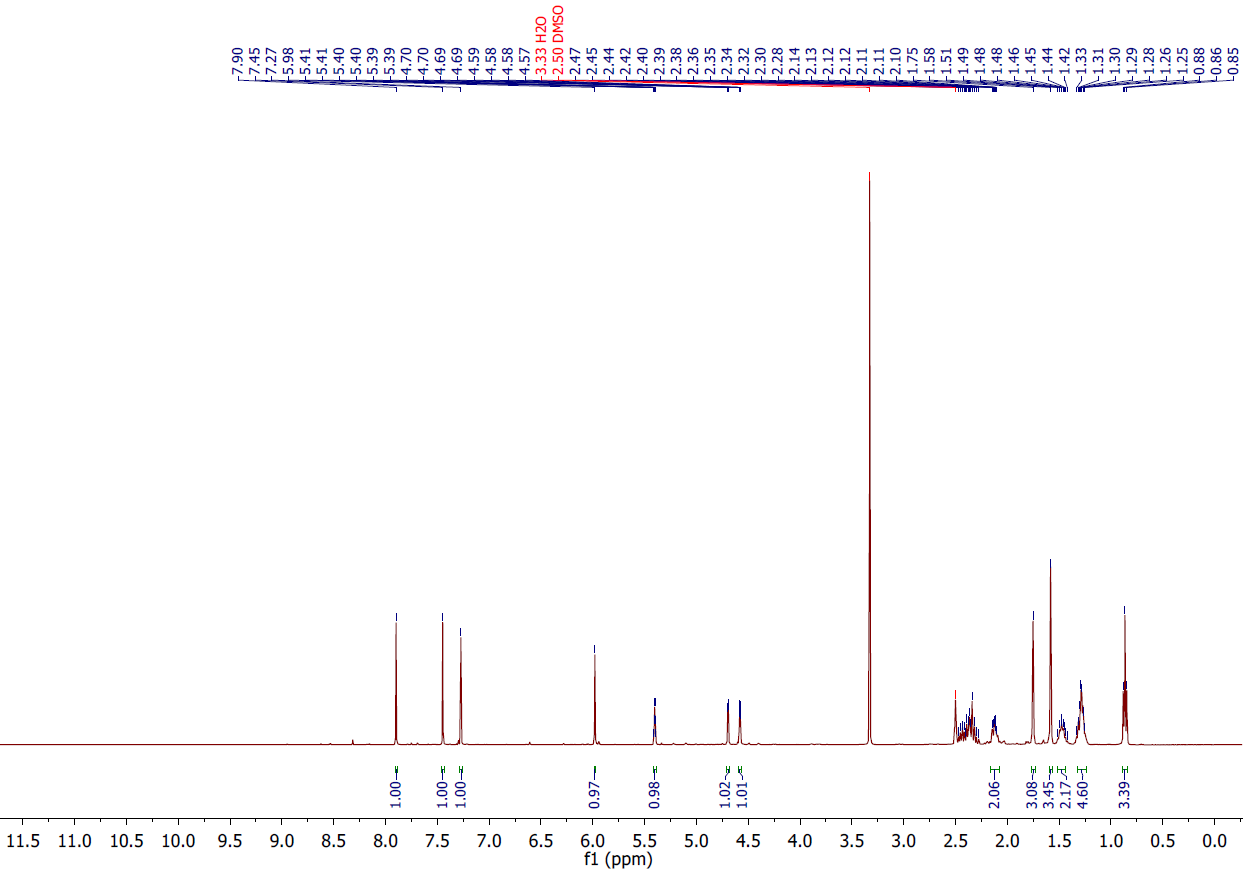


**Figure S29.** Hydroxyquinol **5** ^13^C{^1^H} NMR (400 MHz, DMSO-*d*_6_)

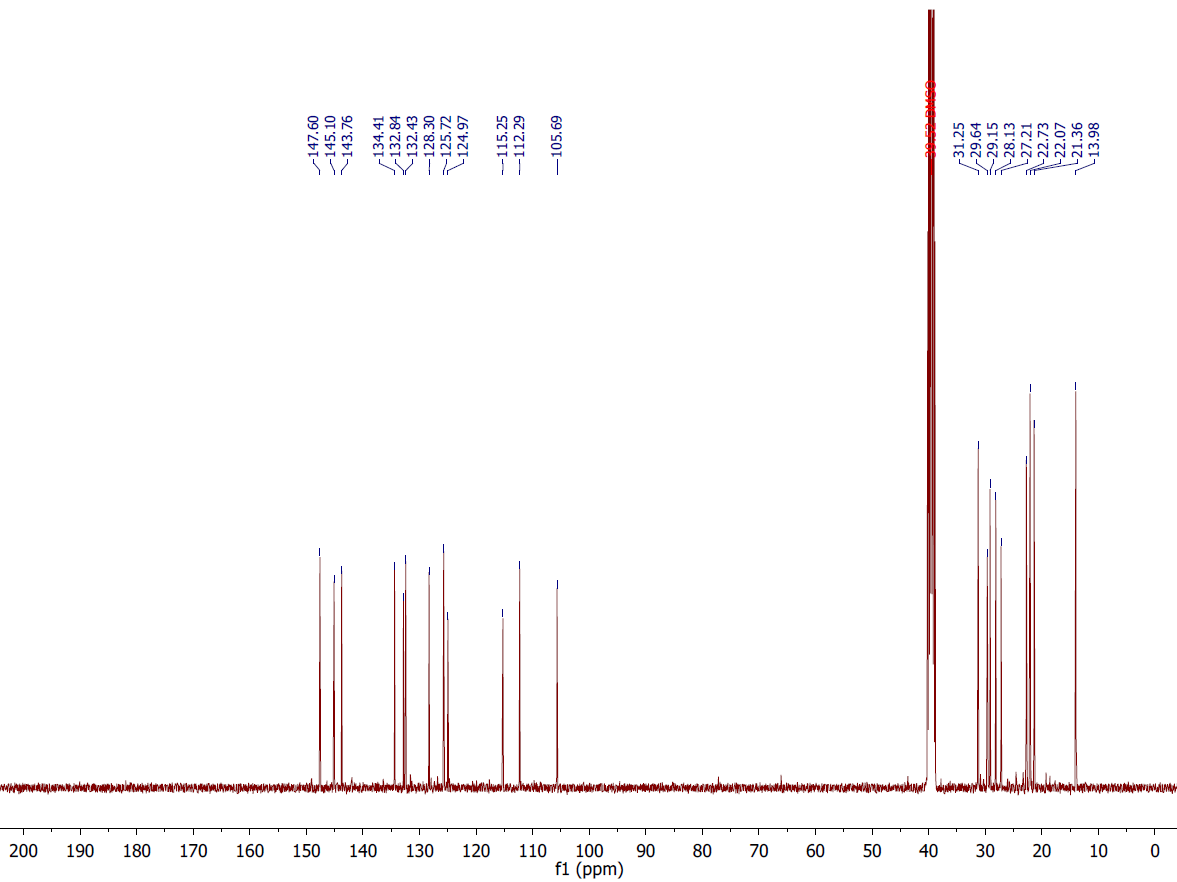


**Figure S30.** Hydroxyquinol **5** HSQC (400 MHz, DMSO-*d*_6_)
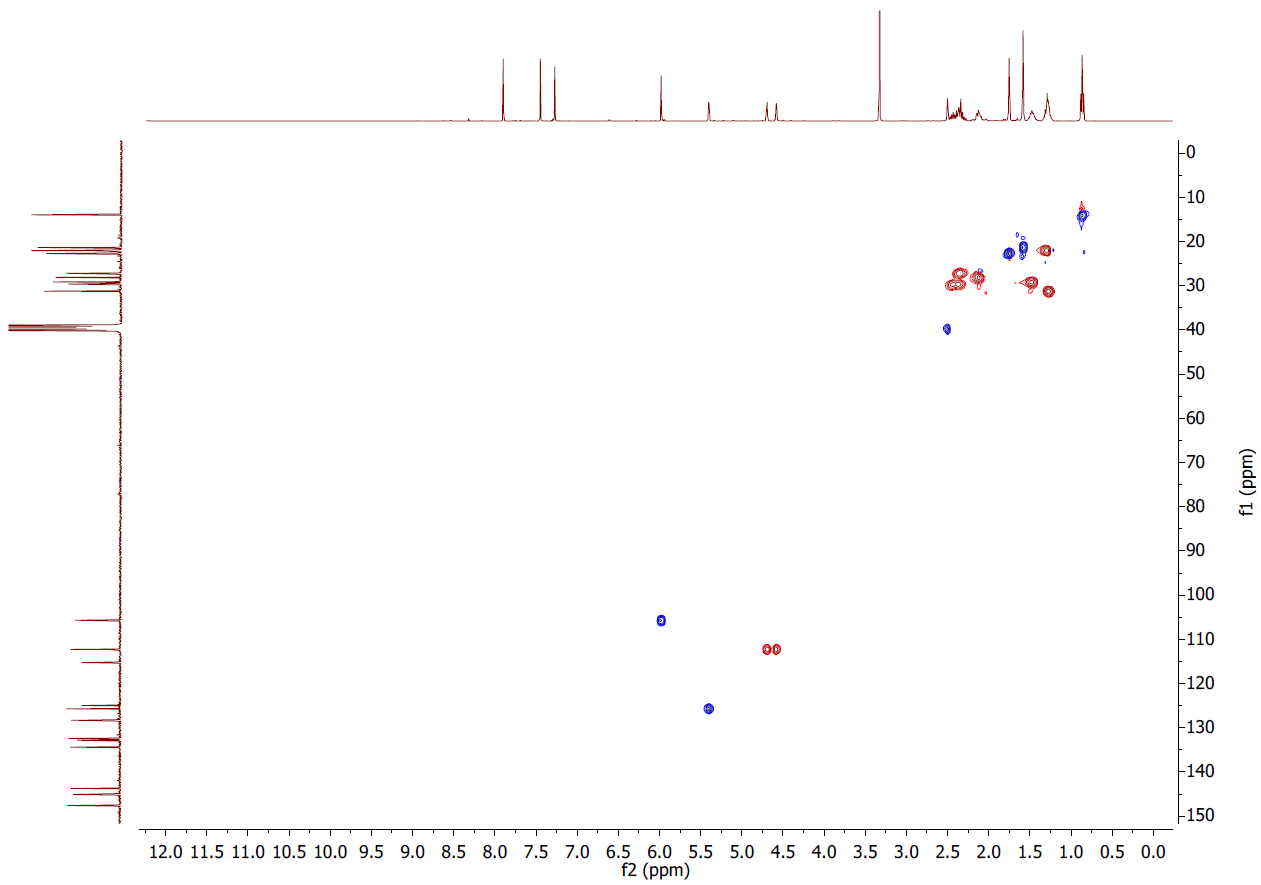


**Figure**
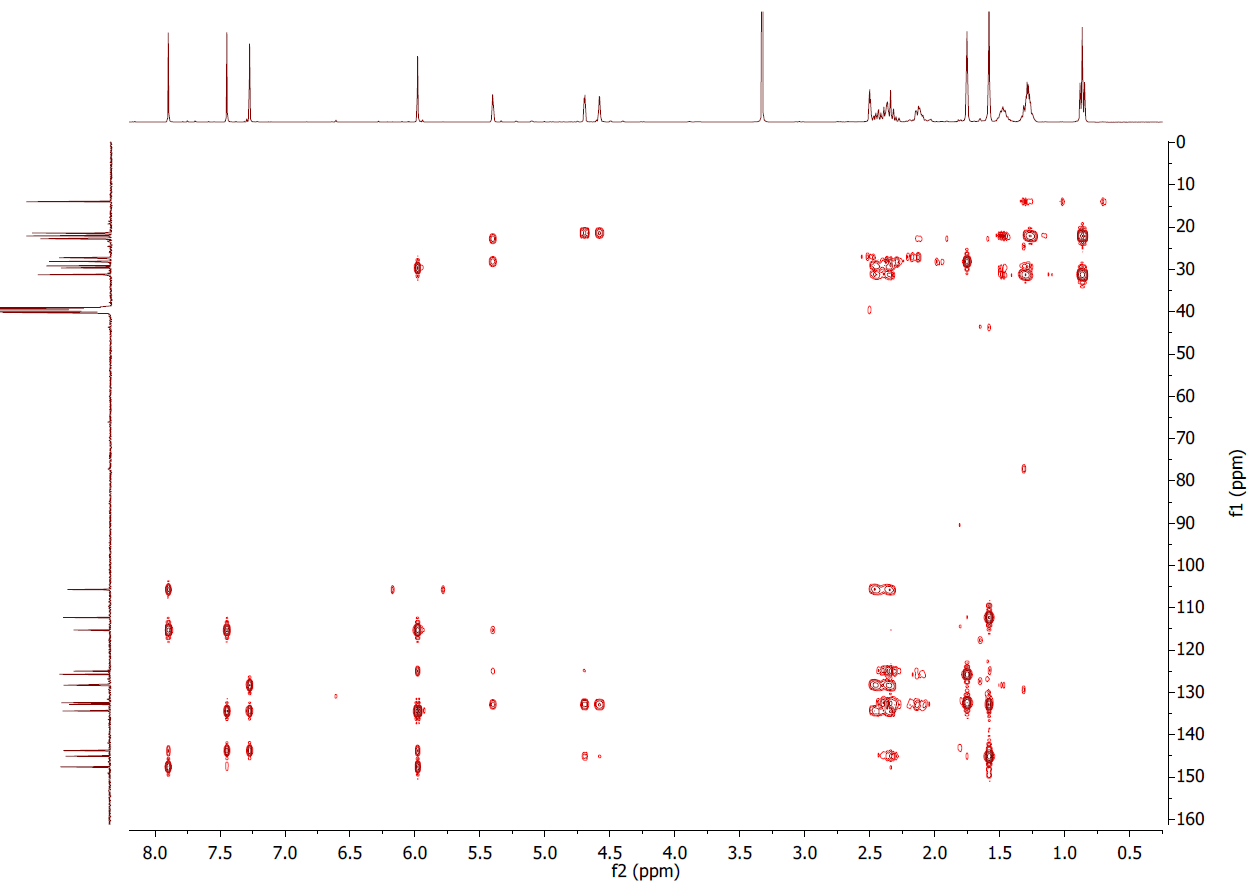
 **S31.** Hydroxyquinol **5** HMBC (400 MHz, DMSO-*d*_6_)

## Unknown contaminants of aged CBD samples

**Figure S32:** Example chromatogram of 27aged samples of isopropanol containing CBD and HU-331.


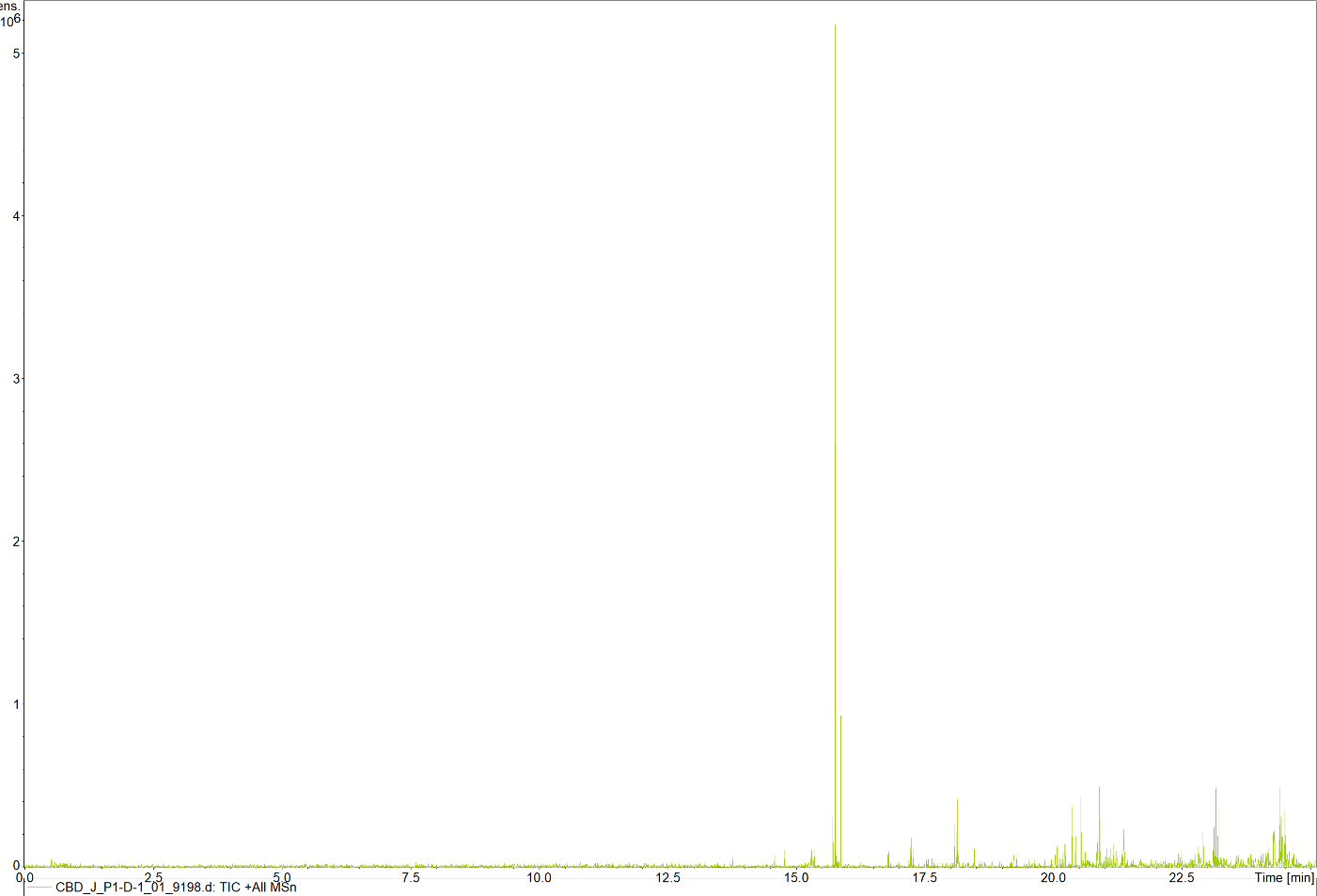


## Colour changes of HU-331 solutions after exposure to light


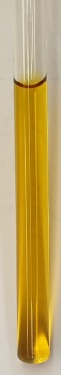

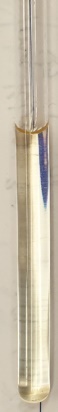

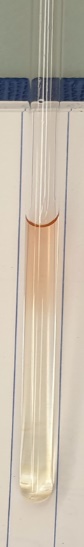

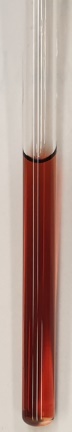

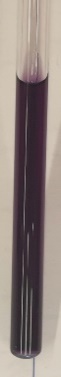


**Figure S33:** Colour changes of HU-331 after photoexcitation with 448 nm light. HU-331, hydroxyquinol **6**, hydroxyquinol **6** after exposure to oxygen for 1, 24, and 48 hours (left to right).

## Identification of H_2_O_2_

To a 1 mL vial was dissolved HU-331 (**2**) (2 mg, 0.006 mmol) in DMSO-*d*_6_ ­(0.6 mL) before being subject to 448 nm of light described by the general experimental setup for 15 minutes. The solution was split between 2 NMR tubes before both were diluted with additional DMSO-*d*_6_ (0.3 mL) and one by spiked with 30 % H_2_O_2_ before being analyzed by ^1^HNMR spectroscopy.

### Supporting NMR spectroscopic data

**Figure S34.** Hydroxyquinol **5** formation before H_2_O_2_ spike. ^1^H NMR (300 MHz, DMSO-*d*_6_)


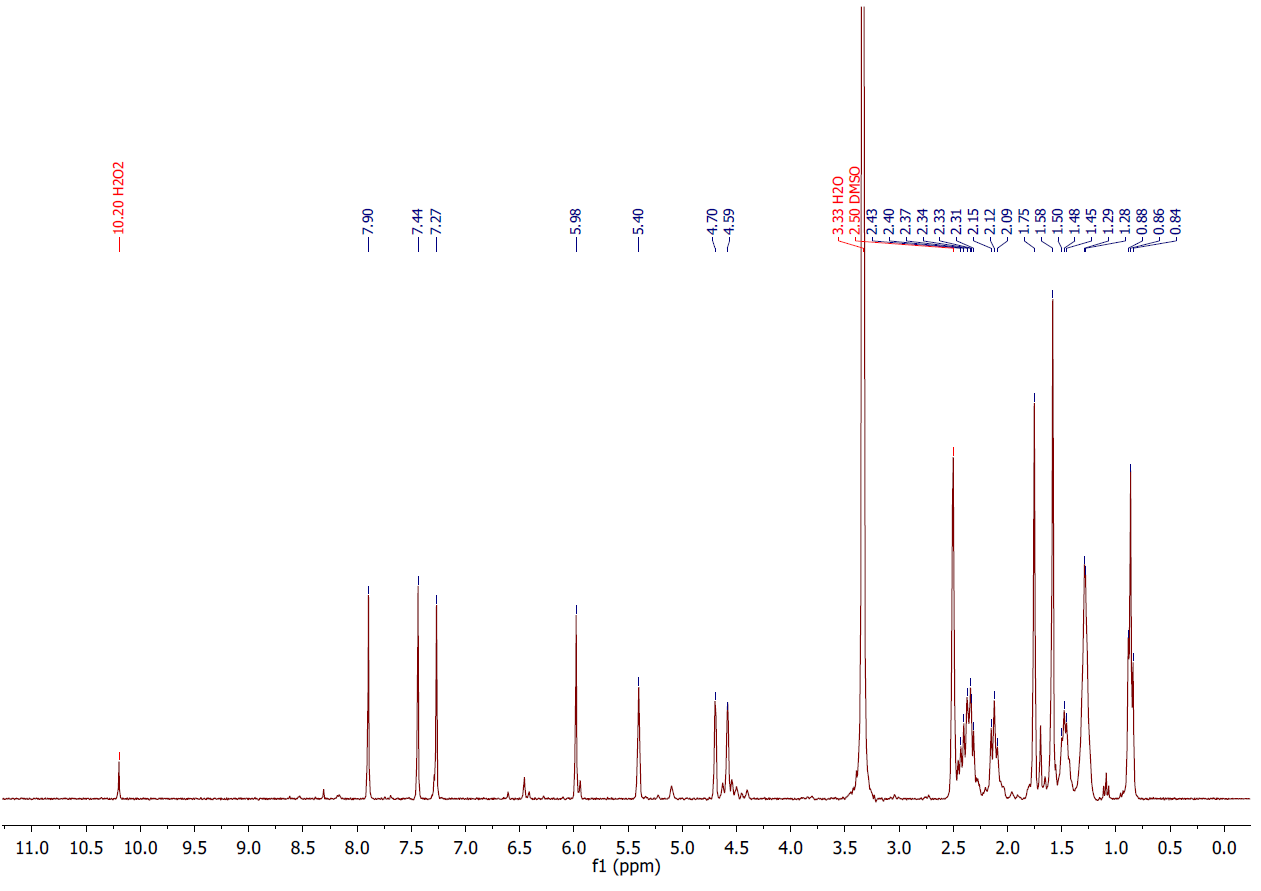


**Figure S35.** Hydroxyquinol **5** formation after H_2_O_2_ Spike. ^1^H NMR (300 MHz, DMSO-*d*_6_)
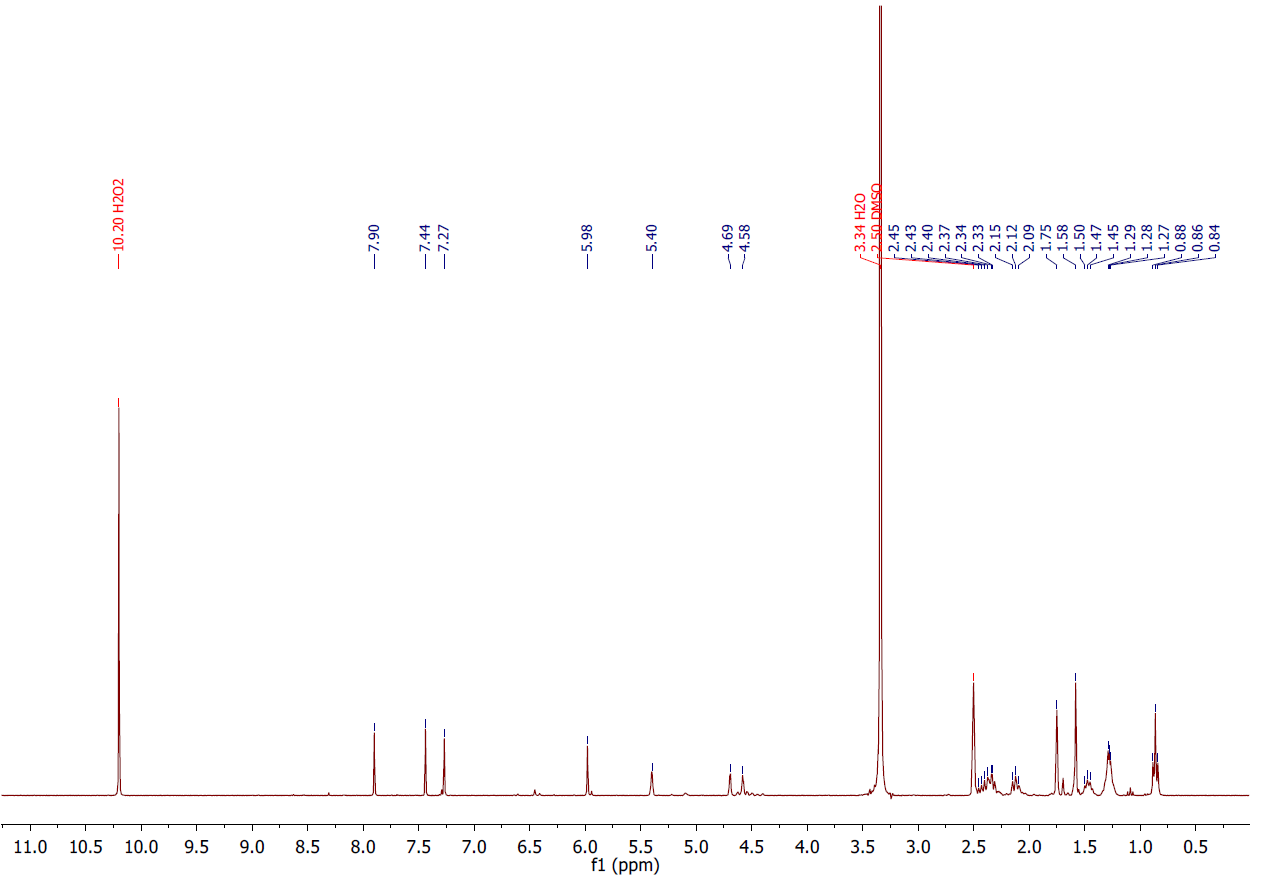


##

## Determination of additional quinones in solutions of CBD (**1**)

Isopropanol standard solutions (1 mL) containing CBD (**1**) (0.5 mg mL^-1^) and/or HU-331 (**2**) (0.5 mg mL^-1^) were left in natural sunlight for 13 days. Aliquots were removed for analysis by LC/MS.

**Figure S36.** Characteristic molecular fragment structures used to screen QTOF data for matches.

### Table S2: Example matches discovered in degraded CBD solutions suitable for follow-up study

**Table S2.** Example matches discovered in degraded CBD (**1**) solutions suitable for follow-up study

| RT (min) | Precursor m/z | Reference m/z | Formula | RT matched |
| --- | --- | --- | --- | --- |
| 12.81622 | 343.1901 | 343.19 | C21H26O4 | TRUE |
| 15.31157 | 359.1888 | 359.189 | C21H26O5 | TRUE |
| 15.64638 | 347.2211 | 347.22101 | C21H30O4 | TRUE |
| 15.54622 | 347.2218 | 347.22171 | C21H30O4 | TRUE |
| 14.95592 | 327.1981 | 327.19699 | C21H26O3 | TRUE |
| 12.82322 | 343.1911 | 343.19 | C21H26O4 | TRUE |
| 14.9144 | 331.2301 | 331.229 | C21H30O3 | TRUE |

##

## Olivetol beam test

To an open 250 mL RBF was dissolved olivetol (180 mg, 1 mmol, 1 equiv) in toluene (60 mL). ^t^BuOK (337 mg, 3 mmol, 3 equiv) was charged to the flask and the reaction was left to stir for 6 hours at room temperature. The solution was acidified with HCl before the organic layer washed with sat. sodium bicarbonate and dried over MgSO_4_. Toluene was removed under reduced pressure to afford a red oil. Excess DBU was added to an aliquot of the oil dissolved in IPA before the solution was analysed by UV-vis measuring in the visible range.

^1^HNMR spectroscopy identified olivetol as the primary product (>99 %), suggesting a slowed reaction rate without the terpene fragment. For this reason, the concentration of any formed hydroquinone is unknown. Excess DBU was added to identify any low-concentration species with absorptions around 540 nm. λ_max_ of 520 nm (Fig. S37) suggested the presence of hydroxyquinone anions.

## Visible light absorption comparison between olivetol-HQ and HU-331 anion

**Figure S37:** Visible-range spectrum of olivetol solution after being subject to Beam test conditions (dashed line, Λ_max_ of 520 nm) compared with the visible-range spectrum of HU-331 anion (solid line, 0.5 mg mL^-1^, Λ_max_ of 540 nm).

## Coordinates from ORCA-job HU-331 (**2**)

**Singlet HU-331 (2)**

52

C -5.21106142575376 -2.18339094117980 0.17817788527854

C -3.67232018598622 -2.22211002580547 0.08798793867299

C -3.08504059465650 -0.82933427472652 0.17097317098258

C -3.72661453764170 0.23875904795885 0.63704332273701

C -5.14216779300144 0.14896018457537 1.14249290978179

C -5.60317617975584 -1.28874645494029 1.36492914808032

H -3.29738148730562 -2.78506134993047 0.94653642012134

H -5.57104003985737 -1.69221862607916 -0.72845093937426

H -5.23078781184655 0.71940217093101 2.07347769281970

H -5.80419568192754 0.65081153954098 0.42505532238205

H -5.14533407207941 -1.69394092916233 2.27240183319846

H -6.68376607449914 -1.31610818014386 1.51857416716338

H -2.06211911060824 -0.72451937191114 -0.17743853254792

C -3.09584840505219 1.59887957143183 0.67129178013148

H -3.04853043994485 1.98541555918817 1.69449314672314

H -2.08545530130975 1.58597699119929 0.26168459265332

H -3.68968337077922 2.31433746625282 0.09271631110929

C -3.17589734495881 -2.94749252195329 -1.13885540180319

C -2.35532153523417 -4.01480619030951 -1.05776478958308

C -3.54824667331892 -2.48917492398984 -2.48869523004279

C -1.85690565581154 -4.75703090649332 -2.25031043532965

C -3.05068308160920 -3.23435825196511 -3.67543623078617

C -2.26135936852399 -4.31172506225192 -3.59955924295125

H -3.38180321713068 -2.83412003846425 -4.62667283605890

O -4.25779024193331 -1.51109693918634 -2.67501477397108

O -1.11987288807891 -5.71483852219309 -2.05913759093090

O -1.91548559480671 -4.51898711974267 0.10531857269746

H -1.35428596871887 -5.28310614605025 -0.13089996812552

C -1.78474113553683 -5.10393492282518 -4.77650212993683

H -0.72597242440938 -5.34016597122827 -4.64425258122181

H -1.87865339728875 -4.49452946529836 -5.67805137590971

C -2.56511455731707 -6.41535276616846 -4.95833181167560

H -2.48969069722401 -7.00727646081136 -4.04290331932317

H -3.62547177612504 -6.18135101969243 -5.09710194157120

C -2.05917517335257 -7.23653743414908 -6.14038835064494

H -0.99718685723733 -7.46329663129159 -5.99365300013785

H -2.11942306494730 -6.63539589622429 -7.05513658362401

C -2.82993835196222 -8.53943764593966 -6.34166318678765

H -2.76873986782657 -9.13718993414130 -5.42630678296471

H -3.89128144733743 -8.31109672469925 -6.48569341844611

C -2.31803517100703 -9.35885440095253 -7.52325789913566

H -1.26734408368277 -9.62810091358623 -7.38864981470402

H -2.88567254308995 -10.28355688661781 -7.64390875161459

H -2.39722676082013 -8.79521044419524 -8.45639691521870

C -5.88372434582186 -3.53561536705212 0.26407689110796

C -5.31851455282953 -4.62055689206031 0.78800986068290

H -4.30636747031886 -4.62822298127482 1.16814994724687

H -5.86272134658776 -5.55556518745120 0.84822502017501

C -7.28581109084366 -3.56824733453135 -0.27888200242500

H -7.91138481913579 -2.80335437436374 0.19045976229421

H -7.28345402845661 -3.35242616136000 -1.35179494204146

H -7.75770095471088 -4.53811993868487 -0.12176491715197

## Coordinates from ORCA-job triplet HU-331

52

C -5.08500663734030 -2.27490143575876 0.16510320179414

C -3.54315422652223 -2.17033446929077 0.07615894514684

C -3.09287071741692 -0.72950449267797 0.17955567233029

C -3.83915026677204 0.25999264438055 0.67159790064051

C -5.23988149160495 0.02523972412535 1.16696716092449

C -5.56126059016371 -1.45339737782652 1.36865383310974

H -3.10961822204879 -2.72414752859648 0.91276326923906

H -5.46644883424838 -1.78922174232022 -0.73646860155595

H -5.39064076560786 0.57675644529130 2.10072196112829

H -5.94034106950848 0.46559006775751 0.44600941584208

H -5.06913772102450 -1.82871950648511 2.27094536844855

H -6.63461680282876 -1.58588936667378 1.51644851142440

H -2.08704754663437 -0.51683750238446 -0.16564257040678

C -3.33332634367441 1.66800948838901 0.75129072402590

H -3.32596158667285 2.02533688003480 1.78576378285570

H -2.32596139510652 1.76054396563461 0.34579513812979

H -3.99108320827784 2.34062399768948 0.19094883904879

C -3.03441955508199 -2.78971322148770 -1.18364481689992

C -2.13929205360569 -3.90921274008068 -1.14771606056142

C -3.47669239963803 -2.34239051999597 -2.49454236764453

C -1.75741603089604 -4.68887461388811 -2.29896223671103

C -3.07747375110745 -3.15036337297864 -3.62830730180804

C -2.26831480378164 -4.25585019068133 -3.56939812071008

H -3.47654650281704 -2.82518373055584 -4.58202409181500

O -4.23678594086967 -1.35859018622070 -2.62009237347282

O -0.96560378878800 -5.64874771909812 -2.08818900451515

O -1.55610823405017 -4.27330437956780 -0.02594907160774

H -1.00615824469287 -5.05459583926778 -0.31854754778438

C -1.89484862058966 -5.04526104325142 -4.78554324637509

H -0.82788124140153 -5.27916121117473 -4.74163123813240

H -2.07044362690508 -4.44624403002820 -5.68190094535847

C -2.67981022316596 -6.36382470773799 -4.88158247560309

H -2.51596051683650 -6.93907444876789 -3.96721724814197

H -3.75025279971141 -6.13873941571484 -4.92829604983312

C -2.27512797092967 -7.19865016001133 -6.09269297047183

H -1.20288187262668 -7.41748818819939 -6.03680417272747

H -2.41977645994974 -6.61136455022954 -7.00715234270273

C -3.05162311224945 -8.50868297120742 -6.20705343077745

H -2.90673713709843 -9.09186789689919 -5.29172412983936

H -4.12312802719906 -8.28844574599032 -6.26131806302886

C -2.63940777412026 -9.34358864940142 -7.41659741544638

H -1.57914232210713 -9.60493096716116 -7.37006229293124

H -3.20914735955411 -10.27326741907513 -7.47411713873440

H -2.80297752689734 -8.79444934311474 -8.34749574866240

C -5.61679537809941 -3.68917899945680 0.18121730181091

C -4.93140308183414 -4.74184397872829 0.62552884899868

H -3.92631787977241 -4.66480397723103 1.01534148934734

H -5.36995660814279 -5.73206828450876 0.62229106203382

C -7.01441474016319 -3.83250900092836 -0.35270330676814

H -7.70842368201162 -3.16657900010732 0.16776971130965

H -7.04516010075811 -3.55122576012766 -1.40955260554915

H -7.38176958709448 -4.85347003841266 -0.25321337101236

## Calculated orbital diagrams

**Figure S38.** Orbital Pictures (0.05 iso)


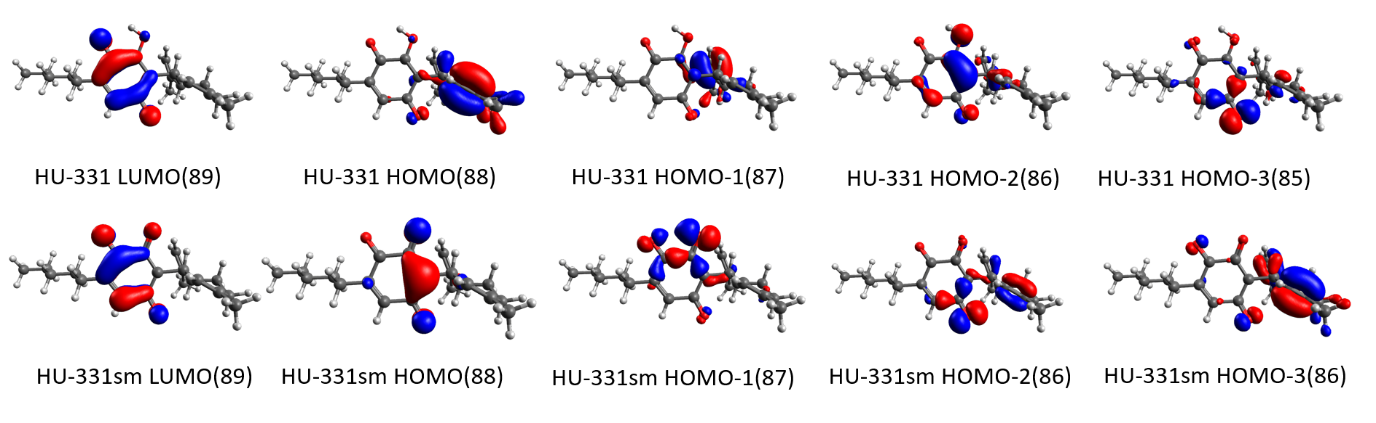


# References

1. Kogan, N. M.; Rabinowitz, R.; Levi, P.; Gibson, D.; Sandor, P.; Schlesinger, M.; Mechoulam, R. Synthesis and Antitumor Activity of Quinoid Derivatives of Cannabinoids. *J. Med. Chem.* **47**, 3800-3806; [10.1021/jm040042o](https://doi.org/10.1021/jm040042o) (2004).
